# Proximity Interactions in a Permanently Housed Dairy Herd: Network Structure, Consistency, and Individual Differences

**DOI:** 10.3389/fvets.2020.583715

**Published:** 2020-12-07

**Authors:** Kareemah Chopra, Holly R. Hodges, Zoe E. Barker, Jorge A. Vázquez Diosdado, Jonathan R. Amory, Tom C. Cameron, Darren P. Croft, Nick J. Bell, Edward A. Codling

**Affiliations:** ^1^Department of Mathematical Sciences, University of Essex, Colchester, United Kingdom; ^2^Writtle University College, Chelmsford, United Kingdom; ^3^School of Life Sciences, University of Essex, Colchester, United Kingdom; ^4^Centre for Research in Animal Behaviour, College of Life and Environmental Sciences, University of Exeter, Exeter, United Kingdom; ^5^Royal Veterinary College, Hatfield, United Kingdom

**Keywords:** animal group, animal movement, dairy cow, lameness, local positioning system (LPS), precision livestock farming (PLF), proximity interactions, social network analysis (SNA)

## Abstract

Understanding the herd structure of housed dairy cows has the potential to reveal preferential interactions, detect changes in behavior indicative of illness, and optimize farm management regimes. This study investigated the structure and consistency of the proximity interaction network of a permanently housed commercial dairy herd throughout October 2014, using data collected from a wireless local positioning system. Herd-level networks were determined from sustained proximity interactions (pairs of cows continuously within three meters for 60 s or longer), and assessed for social differentiation, temporal stability, and the influence of individual-level characteristics such as lameness, parity, and days in milk. We determined the level of inter-individual variation in proximity interactions across the full barn housing, and for specific functional zones within it (feeding, non-feeding). The observed networks were highly connected and temporally varied, with significant preferential assortment, and inter-individual variation in daily interactions in the non-feeding zone. We found no clear social assortment by lameness, parity, or days in milk. Our study demonstrates the potential benefits of automated tracking technology to monitor the proximity interactions of individual animals within large, commercially relevant groups of livestock.

## Introduction

The herd social structure of cows on most commercial dairy farms differs significantly from their wild counterparts (1). Dairy cows are typically kept in exclusively female groups, separated by age and reproductive status, with access to a more restricted space allowance in the form of either indoor housing or fenced grazing paddocks and may be subject to frequent regrouping events (2–5). Understanding the structure and dynamics of housed dairy cattle networks may give insights on preferential interactions and aid in optimizing their management (6, 7).

The social structure of animal groups, including how associations and interactions between individuals change over time, can be assessed using social network analysis (SNA) (8). The approach is well established; SNA is used across multiple disciplines including sociology (9), computer science (10), and transport (10, 11), and has been developed to study animal social networks, particularly over the last decade (12, 13). SNA has been used to explore interactions in dairy cattle, revealing highly clustered herds (14–16). Cows appear to associate non-randomly, potentially based on attributes such as lactation number (14, 15). Inter-individual variation in sociality has been found in dairy cattle, potentially driven by personality, established as consistent from calf to adulthood (except during puberty) (17), or dominance, as studied in (18) who found that some individuals are more influential than others within the social network. Housed cattle are known to avoid interactions with dominant conspecifics whilst feeding to reduce competition (19), and the social positioning of individuals may also be altered where a resource is deemed more valuable (20). Individual attributes are thought to be important in disease transmission (7), as cows participate in contact behaviors based on age and sex. Dairy cows may groom conspecifics based on familiarity and dominance (21), although affiliative and agonistic interaction networks may not be correlated (22).

Data can be collected for SNA in non-automated ways, such as through direct observation (7, 21) or through analysis of video recordings (22). Although detailed social interaction data can be obtained through these methods, they are highly time-consuming, and limit sample size and sampling duration. Developments in technology mean that it is now possible to collect absolute or relative spatial positioning data in an automated way using proximity sensors or positioning systems, recording detailed locations of all animals in the herd over time. Global positioning system (GPS) can be used to track cattle outdoors (23), but mean location errors are typically around 5 m in commercial systems and can be as high as 19.6 m (24). As GPS does not function indoors, alternative systems are needed for housed dairy cows, such as sensor-based local positioning systems (LPS), which have been validated with dairy cows with mean error typically around 2–3 m, although 0.5 m mean error may be achievable (15, 25–28). The simplest interaction networks are then developed by assuming interactions occur when two individuals are within a given proximity, usually based on metric distance, for a specified time duration (6, 8, 14, 29); while analysis based on topological distances (30) or more complex interactions and social dominance are also feasible (31).

Modern productions systems, while efficient, expose cattle to risks for several production diseases, including lameness, mastitis, and metabolic diseases. Lameness is a significant issue globally with average farm level prevalence estimates of 28–32% in Europe (32, 33), 28–39% in South America (34, 35) and 30–55% in North America (36). System related promoters of lameness include high yields (37, 38) driven by genetic selection, and nutrition and environmental factors such as increased standing time on unsuitable floor surfaces (39–41). Early detection of lameness and prompt treatment is essential to reduce its severity and duration (42, 43) and to prevent re-occurrence (44, 45). Under-estimation of lameness by farmers remains a problem which can lead to delays in treatment (46–48). To identify lame cows, farmers typically observe elements of a cow's gait, which is prone to error and largely subjective (49), and abnormal behaviors may not be immediately obvious (50). While on some farms this process may be formalized by scoring all cows against a recognized locomotion score (51), on many farms cows are only observed during routine tasks, increasing subjectivity and the risk of missing a large proportion of the herd. Precision Livestock Farming (PLF) techniques, where farm management is aided through continuous automated real-time monitoring of animals or the environment (24, 26, 27) provide opportunities to support rapid identification of lameness and other production diseases. Lameness has been associated with inflammatory responses (52) and results in generalized sickness behaviors which could be monitored using PLF techniques. Changes to individual cow behavior associated with lameness have also been investigated using PLF techniques to identify modified feeding and lying behavior (53–56), and space use (57). Sick cows are less likely to approach humans (58, 59), and both cows and calves have been observed to alter their positioning in a herd when ill (60–62). Evidence suggests cows with ketosis and mastitis displace conspecifics less frequently (63–65). Lame cows may alter their time budgets with lame individuals spending less time feeding than their healthy counterparts (53, 57). Lame cows also appear to be licked by conspecifics more than non-lame cows (66). Despite this existing evidence, to our knowledge automated PLF techniques have not been applied to monitor changes in social behavior in cattle that could be associated with disease.

In this study we investigate the structure and consistency of the proximity interaction network of a large permanently housed dairy herd using positional data collected from an automated local positioning system (LPS). We determine the level of inter-individual variation in proximity interactions across different functional zones of the barn (feeding, non-feeding) and assess how these interactions vary during the month-long study period. We consider the influence of health status (specifically lameness), parity, and days in milk (DIM), on the sociality and interactions of individuals within the herd.

## Methodology

### Animals and Housing

**Figure 1:**
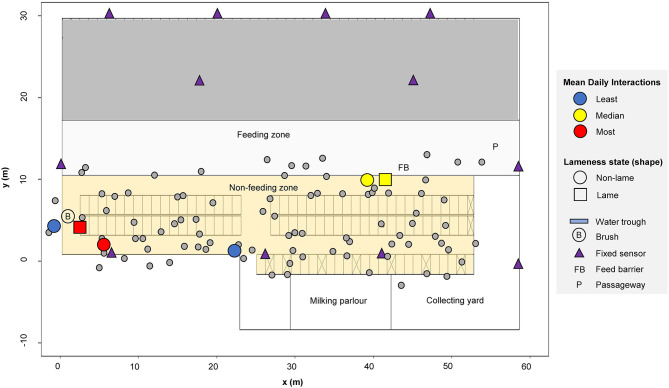
Barn layout showing (*n* = 92) example cow locations recorded on 01/10/2020 at 05:09:50. A highlighted illustrative subset of cows (*n* = 6) are colored according to their mean daily interactions: least = blue (cow ID = 3124 and 3317), median = yellow (cow ID = 3602 and 3132), most = red (cow ID = 635 and 3361). Data for each individual cow is indicated with a small circle. The area shown in gray (upper barn area) was not used by this group of cows during the study period.

A high-yielding management group of Holstein-Friesian dairy cattle were observed continuously throughout October 2014 on a commercial farm in southeast England. Our study group consisted of 92 cows that were continuously present in the barn throughout the study duration (mean days in milk (DIM) = 136 and mean parity = 3). These cows formed part of a larger group (100 to 111 on any given day in the month, mean = 105, standard error = 0.59), with averages calculated from April 2013 to April 2014 of: calving interval of 416 days, 305 daily milk yield of 10,909 liters, 63% pregnant, somatic cell count of 140,000 cells/ml. Localized weather and temperature, which are known to affect behavior (67), were largely stable throughout the study period (mean range of 12.4–19.9 degrees Celsius). Cows were housed permanently indoors inside one half of a commercial free-stall barn containing 98 useable cubicles bedded with sawdust over mattresses (Figure 1). Central passageways allowed free movement around the barn and access to the central feeding passage. Cows were milked three times per day (morning, 5 a.m.; afternoon, 1 p.m.; and evening, 9 p.m.) and provided with a total mixed ration once daily during morning milking; fresh feed was pushed up several times throughout the day. Health status, parity and days in milk were downloaded from the farm records, held in UNIFORM- (UNIFORM-Agri, Somerset, UK). A specific study of the effects of lameness on behavior with a smaller subset of the same herd group, within the same barn environment but over a different time period, has previously been reported (53, 57).

During the study, cows were assigned a mobility score fortnightly as they exited the parlor (on the 30/9/2014, 13/10/2014, and 27/10/2014), using the AHDB mobility score (51). A mobility score of 0- 3 was assigned, where 0 is good mobility, 1 is imperfect mobility, 2 is impaired mobility and 3 is severely impaired mobility (Supplementary Material 1). If a score was not recorded, “NS” was noted. For this study, cows with score 2 or 3 were considered as clinically lame (L) and cows with scores 0 or 1 were considered non-lame (NL). Cows scored as not lame for two successive scoring sessions (NL-NL-L or L-NL-NL) were classed as “dominant not lame,” and cows scored as “dominant lame” for most sessions (L-L-NL or NL-L-L) were classed as lame. Cows that changed status twice within the study (NL-L-NL or L-NL-L) or those with missing data were not included in the lameness classification. For the purposes of the main analysis presented here, we combine “lame” and “dominant lame” cows into a single group (“lame”), and similarly “non-lame” and “dominant non-lame” cows are combined into a single group (“non-lame”). In total, 48 of the 92 cows within the study group were classified as either “lame” (22 cows) or “non-lame” (26 cows) using this approach (Supplementary Material 1), and were included in the part of our analysis focusing on lameness differences. Our results are qualitatively similar if we do not combine the groups and keep four separate classifications for lameness, see Supplementary Material 1.

### Local Positioning System

Cows were each fitted with a mobile Oms500 (Omnisense Ltd, Cambridge, UK) combined local-positioning and accelerometer sensor, attached to a weighted neck collar to ensure the sensors remained stable in the same orientation. The sensors deployed on the cows form a localized wireless network that uses triangulated radio signal communication to automatically determine the relative local position of every cow in the herd, at a temporal resolution of 0.1 Hz throughout the full study duration. Additional fixed sensors were strategically positioned throughout the barn to fix the absolute spatial location of each sensor and to maximize the sensor network performance (Figure 1). The performance of this specific sensor system in the same barn environment was evaluated in (53), who reported a 50% circular error of probability (CEP) measurement of 1.07 m for a static sensor (not mounted on a cow) and 1.90 m for a sensor mounted on a standing cow (i.e., 50% of all measurements lay within 1.07 m of the mean location of static sensors and within 1.90 m of the mean location of cow mounted sensors). In the same study, mean distance errors of 2.66 m (static sensors) and 2.80 m (sensors on standing cows) were also reported.

### Pre-processing and Cleaning of Positional Data

All data processing and analysis was conducted in R for Windows 3.6.3 64 bit, with RStudio (68, 69). Extended interruption occurred because of a system malfunction on three of the study days (09/10/2014, 27/10/2014, and 31/10/2014); these incomplete days were not included in the analysis. Data cleaning and analysis were conducted on the 92 cows which were continuously present in the free-stall barn throughout the study duration (*d* = 28), see Supplementary Material 2 for full details. In the first pre-processing step, location data further than a 3 m buffer distance outside the main barn area were removed; the 3 m buffer was included to avoid excluding data due to minor positional inaccuracies. Data removed at this stage included (correct) locations recorded in the milking parlor and collecting yard (where the cows were constrained for up to 3–4 h per day in total during the three milking events), as well as (incorrect) erroneous locations entirely outside the barn area. In total 22.81% of the original data was removed in this step. An automated “cleaning” algorithm was then used to identify and remove any nonsensical positional data (e.g., sensors apparently getting “stuck” in exactly the same, or a similar, point location for several consecutive time points, often shortly after the system reset at the end of each day; 3.06% of original data removed). The remaining location data were smoothed to remove noise using a simple moving average with a window size of 15 time points (corresponding to 150 s; 0.17% of original data removed due to losing 7 points at the start and end of the time series because of the smoothing window). A final combination of automated cleaning, and manual observation and checking, were then used to remove any further nonsensical data identified (e.g., cows that stayed relatively stationary for most of an entire day; 0.01% of original data removed). In total, 26.05 % (5,675,319 points) of the total original data points were removed through these pre-processing and data cleaning stages (see Supplementary Material 2); a total of 16,114,423 data points remained for the subsequent analysis.

### Protocol for Determining Proximity Interactions

Using the smoothed and cleaned positional data, an interaction was defined between dyads (each pair of cows) using a protocol based on sustained proximity (radial metric distance) over a specified time period, and was hence non-directed (if cow A is close to cow B, then B is close to A, and so on). In Supplementary Material 3 we explain how and why we selected a “strict” protocol for identifying proximity interactions. The protocol specifies that, for a given dyad, all inter-cow distances over a time period of *t* = 60 s (i.e., 6 time points at 0.1 Hz) must be contained within a radius of *r* = 3 m for an interaction to be identified. While this parameter choice is consistent with previous studies (e.g., 14,16), we also considered a range of other parameter values for *r* and *t*, as well as less stringent protocols (where only a certain percentage of points within the specified time period need to be within the radius for an interaction to be identified). Using observed data of (*n* = 35) known proximity interactions we were able to validate our algorithm and determine the sensitivity (true positive rate) of this protocol (0.83); it was not possible to estimate the specificity using this observed data, but the *r* and *t* parameters were chosen to reduce the expected false positive rate, as well as taking into account practical and biological considerations, including the sensor mean error distance and the typical size of a dairy cow (see Supplementary Material 3 for details). It should also be noted that qualitatively similar results were obtained when using *t* = 40, 80, 100 s (for *r* = 3 m) (Supplementary Material 3 Tables 6–8) and *r* = 1, 2, 4 and 5 m (for *t* = 60 s) (Supplementary Material 3 Tables 2–5), and hence our conclusions should be robust to this parameter choice.

Positional data within the barn were filtered by coordinate into functional zones: the “feeding zone” (defined as the feeding passage and nearest passageway; see 10.5 m ≤ y ≤ 17.2 m in Figure 1), the “non-feeding zone” (cubicles and passageways; 1.62 m ≤ y ≤ 10.5 m,−1.6 m ≤ x ≤ 58.6 m in Figure 1) and the “full barn” (the combined feeding and non-feeding areas); a buffer of 3 m was used around each zone. The proximity protocol defining an interaction described above was subsequently applied to the data for every given dyad located in each functional zone, outputting the total number of interactions over the course of each day. A non-directed weighted matrix for every given day (*d* = 28) and functional zone was produced, holding the number of interactions recorded for every possible dyad (92 x 92). The matrices were therefore symmetrical, with “NA” inputted along the diagonals of each.

### Network Visualization

The interaction matrices for each day, for the full barn, and each functional zone, were converted into network graphs, using the package “igraph” (70) in R (68, 69), where nodes represent individuals (*n* = 92), and edges represent interactions between dyads, with increasing weight (more interactions) indicated by increasing width of the edges. The Fruchterman-Reingold layout algorithm was used to determine the node positions; connected nodes are pulled toward each other and unconnected vertices are repelled.

### Social Network Analysis

The edge density, the proportion of direct ties in a network relative to the total ties possible, was calculated for the full barn and functional zones (feeding and non-feeding zone). Cows periodically entered and left the feeding zone, so edge density was expected to be lower in this zone, in comparison to the non-feeding zone. The networks were also assessed for components, to reveal any potential divisions or isolated individuals, which could be linked to social assortment by lameness (or other factors) in later analysis.

Permutations are used to test the normality of observed network data and are essentially a form of null model (71, 72). A widely used method to account for the non-independence of dyads in SNA is by using a node-level permutation (71, 72). Node identities are randomized, and the original test statistic is compared against permuted test statistics. Here we implement node-level permutations to test our hypotheses by randomizing the identity of cows (*q* = 10,000 in equation 1). A test statistic, comparing a given measure, i.e., differences in daily interactions between lameness states etc., was calculated for each permutation (*t*_*p*_). If the proportion of permuted test statistics was equal to or more than the original test statistic (*t*_*o*_), was ≥ 5% (*p* ≥ 0.05) (see equation 1), then the null hypothesis was accepted i.e., there was no significant difference in the measure between the groups. A Bonferroni correction was applied to the *p*-value to account for multiple comparisons on the same dataset. As computing an exact *p*-value is not possible with a finite number of permutations, if the *p*-value was calculated to be zero a biased estimator was applied: one was added to both the numerator and denominator of Equation 1, following the suggestion in (73).

(1) $$\begin{array}{l} p=\frac{\;\Sigma \;\left(t_{p}\geq t_{o}\right)}{q} \end{array}$$

#### Social Differentiation

As the data on daily interactions was found to be not normally distributed (Shaprio-Wilk normality test; W = 1.00, 1.00, 0.98, *p* = 0.04, < 0.01 and < 0.001, for the full barn, feeding zone and non-feeding zone, respectively), a Kruskal-Wallis Rank Sum test was conducted to assess whether there is a significant difference in the median daily interactions individuals had, with 10,000 node-level permutations to account for non-independence of dyads.

The interactions between each dyad may be uniformly distributed across an interaction matrix for a given day, or specific dyads may interact more or less than other dyads. The structure of a network can be assessed by comparing the number of observed interactions between every given dyad with the number of expected interactions between every dyad. To assess whether associations between individuals were more heterogeneous than we would expect given a null hypothesis that all dyads associate uniformly, the following statistic for social differentiation (S) was calculated (see Equation 2) based on (29), Appendix 9.4, and following (14):

(2) $$\begin{array}{l} S=\frac{\sum_{i}^{n}\;\sum_{j}^{n}{\left(O_{ij}-E_{ij}\right)}^{2}}{n\left(n-1\right)} \end{array}$$

As shown in Equation (2), the difference between the observed number of interactions and the expected number of interactions was summed for each dyad, and then divided by the total number of dyads (*n* = 4186 [= ((92 x 91)/2)]), for each day.

#### Temporal Variation in Sociality

A Kruskal-Wallis Rank Sum test was conducted to assess whether there was a significant difference in median daily interactions between days, for each functional zone, with 10,000 permutations. Pearson's correlation was used to test if temporal variations in daily interactions were correlated across time in each functional zone, and then with mean daily temperature.

To assess whether the network structure was stable or varied over time, seven interaction matrices were created, each holding the average number of interactions between dyads (*n* = 4186) over four consecutive days. Each consecutive network was compared by conducting a Mantel Test (8, 74). The “mantel” function was used, from the “vegan” package in R (75). As the interaction data within the matrices were not normally distributed (as shown through a one-sample Kolmogorov-Smirnov test), a Spearman's Rank Sum test was used to calculate a Mantel statistic *Z*, for each consecutive averaged matrix, with 10,000 permutations and Bonferroni correction applied to account for multiple comparisons. We also completed a similar analysis using shorter- and longer-day partitions, and results were found to be qualitatively similar (Supplementary Material 3 Table 9).

#### Impact of Lameness Status, Parity, and Days in Milk on Sociality

##### Lameness Status

The mean daily interactions between non-lame (*n* = 26) and lame (*n* = 22) cows were compared using a two-tailed Wilcoxon test, with 10,000 permutations

Node degree (the number of immediate neighbors each node in the network has) was compared between non-lame and lame cows. As a cumulative measure, node degree is less prone to sampling error, such as temporal loss of signal of the sensor system, than other measures such as betweenness (the number of shortest paths that pass through a given node), which can change dramatically with removed or missing data (76), so mean node degree was compared between non-lame and lame cows. Local clustering coefficient (the extent to which nodes cluster in a graph, calculated by the proportion of connections a node has with its neighboring nodes divided by the maximum number of connections that could exist in this neighborhood) was also compared between non-lame and lame cows. The mean node-level measures, calculated for each individual over the full study period, were compared between lameness states using two-tailed Wilcoxon tests with 10,000 permutations (Shapiro-Wilk normality test, *p* < 0.01).

A matrix was created, showing the absolute differences in lameness between all dyads (*n* = 1128), as in (16) (e.g., if cow A was lame, a score of 1 was assigned, and cow B was not lame, a score of 0 was assigned, and their absolute difference would be 1). The absolute difference matrix was compared to the original interaction matrix for every given day, using a Mantel test again with Spearman's Rank Correlation Coefficient. Bonferroni correction was applied to account for multiple comparisons (*n* = 28).

##### Parity and Days in Milk

To assess whether parity and days in milk (DIM) affected social assortment, a matrix was created, showing the absolute differences in parity between all dyads (*n* = 4186), as in (16) (e.g., if cow A had a parity of 1, and cow B had a parity of 3, their absolute difference would be 2). An absolute difference matrix for days in milk (DIM) was also created. The absolute difference matrix for a given attribute was compared to the original matrix for every given day, using a Mantel test (as described in Section Lameness Status).

## Results

### Basic Network Measures and Visualization

**Figure 2:**
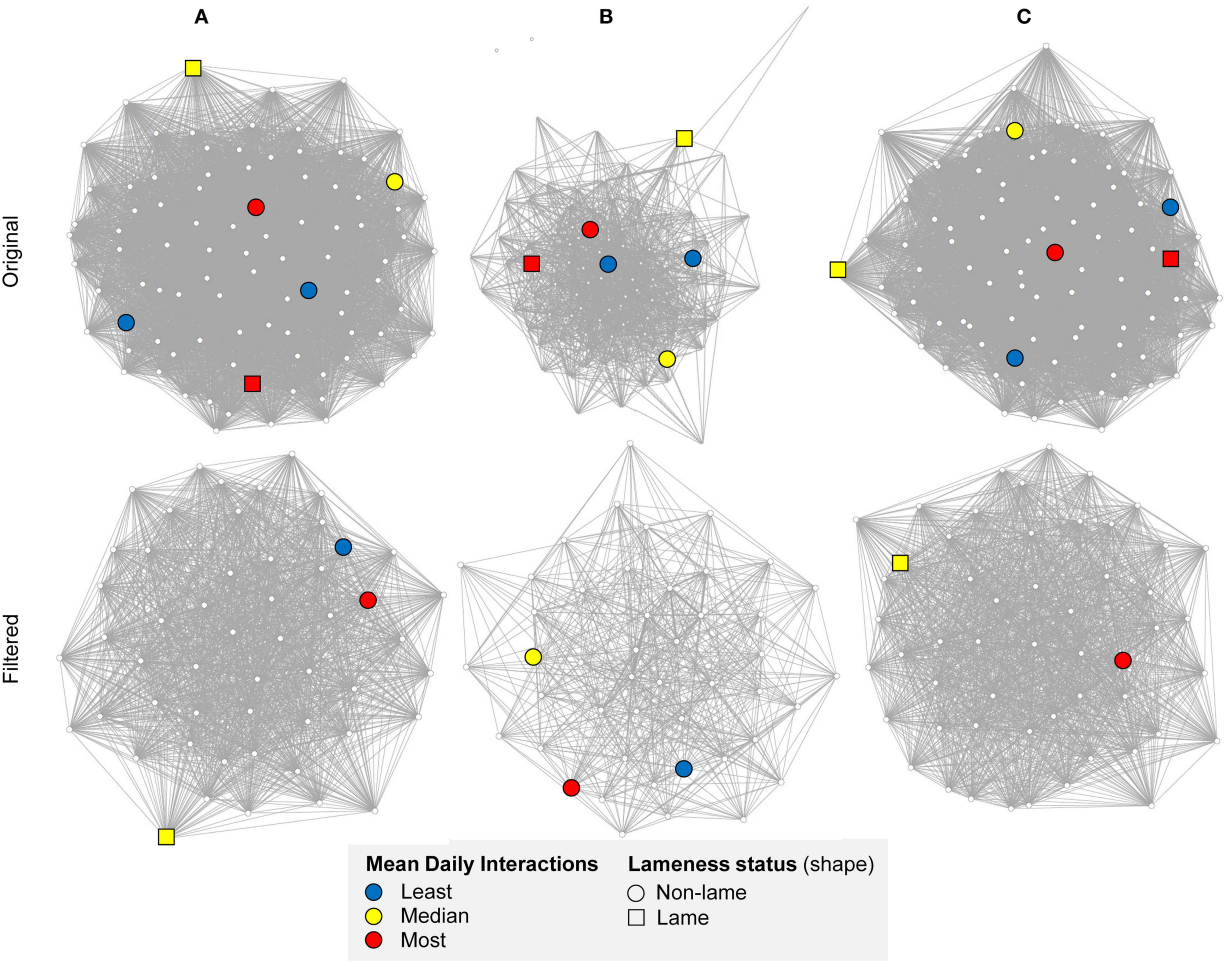
Undirected original and filtered (by mean degree) networks on a randomly chosen day, 01/10/2014, in **(A)** the full barn, **(B)** feeding zone, and **(C)** non-feeding zone, showing mean daily interactions between cows (*n* = 92 in original networks). Thicker edges indicate a higher number of daily interactions. The Fruchterman-Reingold layout algorithm was used to determine the node positions; unconnected vertices are repelled. The highlighted illustrative subset of cows correspond, respectively to the least (blue, cow ID = 3124 and 3317), median (yellow, cow ID = 3132 and 635), and most (red, cow ID = 2273 and 2266) mean daily interactions, with squared nodes representing lame cows. A clearer network structure is shown after filtering, with a more uniform distribution of interactions in the main barn and the non-feeding zone in comparison to the feeding zone. Created in RStudio using the “vegan” package (68, 69, 75).

Figure 2 compares visualizations of the original and mean node degree filtered networks for the full barn, and the feeding and non-feeding zones. A key notable difference between the networks is that the full barn network was more connected than the non-feeding zone network (0.02 difference in edge density) and the feeding zone network (0.63 difference in edge density; Figure 1; Table 1). This is expected since interactions occurring at the boundaries of the feeding and non-feeding zones were likely to be missed when considering these zones separately. The non-feeding zone network was more connected than the feeding zone network (0.31 difference in edge density) (Figure 2; Table 1).

**Table 1:** Overview of results using a spatial threshold radius of *r* = 3 m and time duration of *t* = 60 s to define an interaction for the full barn (FB) and the functional zones: feeding zone (FZ) and non-feeding zone (NFZ): basic network measures (original and filtered by mean degree), inter-individual variation, temporal variation in sociality, lameness status, and parity and days in milk, where (M)DI = (median) daily interactions.

	**Measure**	**Test value (*****p-*****value)**			**Summary**
		**Full barn**	**Feeding zone**	**Non-feeding zone**	
Basic network measures	Mean edge density (*d* = 28)	0.96	0.33	0.94	The networks are highly dense, more so the NFZ than the FZ.
	Components (by day) (*d* = 28)	1	2-6	1	The networks typically consist of one component.
Inter-individual variation	Inter-individual differences in median DI (*n* = 92)	K-W = 26.53 **(*****p<*** **0.001)**	K-W = 851.71 (*p =* 1)	K-W = 19.21 **(*****p*** **<** **0.001)**	**Inter-individual variation in DI in the NFZ** but not in the FZ or the FB.
	Social differentiation (SD) (*n* = 92)	SD between ≤ 92.96 % of dyads **(*****p*** **<** **0.01)**	SD between 100 % of dyads **(*****p*** **<** **0.01)**	SD between 92.96 % of dyads **(*****p*** **<** **0.01)**	**Social differentiation present in all networks**.
Temporal variation in sociality	Difference in median DI between days (*n* = 92, *d* = 28)	K-W = 2252.30 (*p* = 1)	K-W = 61.00 (*p =* 1)	K-W = 2268.9 (*p =* 1)	No difference in DI between days in all networks.
	Relationship between MDI and days (*n* = 92, *d* = 28)	Pearson correlation, ρ = 0.03 (*p* = 0.88)	Pearson correlation, ρ = 0.55 **(*****p*** **<** **0.01)**	Pearson correlation, ρ = 0.02 (*p* = 0.90)	MDI correlated over time in the feeding zone but not in the non-feeding zone.
	Relationship between MDI and temperature (*n* = 92, *d* = 28)	Pearson correlation, ρ = 0.04 (*p* = 0.83)	Pearson correlation, ρ = −0.09 (*p =* 0.66)	Pearson correlation, ρ = 0.04 (*p =* 0.82)	Weak correlation between MDI and temperature in both functional zones.
	Relationship between four-day block consecutive networks (six networks, *n* = 92 per network)	Mantel test, range of R_s_ = 0.03 to 0.23 **(*****p≤*** **0.001)** for three comparisons (day blocks 1–2, 2–3, 5–6); range of R_s_ = −0.04 to−0.001 (*p* > 0.23) for three comparisons (day blocks 3–4, 4–5, 6–7)	Mantel test, range of R_s_ = 0.20 to 0.31 **(*****p*** **<** **0.001)**	Mantel test, range of R_s_ = 0.05 to 0.24 **(*****p<*** **0.01**) for four comparisons (day blocks 1–2, 2–3, 5–6, 6–7); range of R_s_ = −0.04 to 0.01 (*p* = 1) for two comparisons (day blocks 3–4, 4–5)	Weak correlation between all consecutive networks.
Individual characteristics	Difference in mean DI between non-lame (*n* = 26) and lame cows (*n* = 22)	Wilcoxon test, W = 297 (*p* = 0.56)	Wilcoxon test, W = 342 (*p* = 0.86)	Wilcoxon test, W = 276 (*p* = 0.40)	No difference in DI between non-lame and lame cows in both functional zones.
	Difference in mean clustering coefficient between non-lame (*n* = 26) and lame cows (*n* = 22)	Wilcoxon test, W = 392 (*p* = 0.98)	Wilcoxon test, W = 284 (*p* = 0.53)	Wilcoxon test, W = 398 (*p* = 0.99)	No difference in clustering coefficient between non-lame and lame cows in either functional zone.
	Difference in mean node degree between non-lame (*n* = 26) and lame cows (*n* = 22)	Wilcoxon test, W = 304.5 (*p* = 0.63)	Wilcoxon test, W = 321.5 (*p* = 0.25)	Wilcoxon test, W = 241.5 (*p* = 0.17)	No difference in node degree between non-lame and lame cows in either functional zone.
	Social assortment by lameness status by day, *n* = 48)	Mantel test, R_s_ = 0.11 **(*****p*** **<** **0.01)** for day 16; range of R_s_ = −0.07 to 0.05 **(***p =* 1) for remaining 27 days	Mantel test, range of R_s_ = −0.06 to 0.04 (*p =* 1 for all days)	Mantel test, range of R_s_ = −0.07 to 0.06 (*p* > 0.80)	Cows did not socially assort according to their lameness status, parity, or DIM in either functional zone.
	Social assortment by parity (by day, *n* = 92)	Mantel test, range of R_s_ = −0.02 to 0.03 (*p* = 1 for all days)	Mantel test, range of R_s_ = −0.05 to 0.03 (*p* = 1 for all days)	Mantel test, range of R_s_ = −0.02 to 0.03 (*p* = 1 for all days)	
	Social assortment by DIM (by day, *n* = 92)	Mantel test, range of R_s_ = −0.03 to 0.03 (*p* = 0.80 for all days)	Mantel test, range of R_s_ = −0.03 to 0.04 (*p* = 1 for all days)	Mantel test, range of R_s_ = −0.03 to 0.03 (*p >* 0.44 for all days)	

*Significant results (p < 0.05) are in bold*.

The full barn and non-feeding zone networks remained as one component, whereas in the feeding zone network one to three individuals isolated from the main component on each day (Table 1).

### Inter-individual Variation

Throughout the following analysis and presentation of results, a subset of individuals at the middle and extreme ends of the data set are highlighted to aid interpretation and to illustrate the extent of the observed data: two with the lowest mean daily interactions over the full study period (cow ID = 3324 and 3317 with mean daily interactions of 1955 and 1956, respectively), two with mean daily interactions closest to the median (cow ID = 2602 and 3132, with mean daily interactions of 2084 and 2085, respectively), and two with the highest mean daily interactions (cow ID = 635 and 3361, with mean daily interactions of 2266 and 2273, respectively); across the full herd the mean daily interactions were 2093 (median = 2085, standard deviation = 76.63).

There was significant inter-individual variation in daily interactions in the non-feeding zone (Kruskal-Wallis chi-squared [hereafter K-W] = 19.21, df = 91, after 10,000 permutations, *p* < 0.01), but not in the full barn (K-W = 26.53, df = 91, after 10,000 permutations, *p* < 0.001) or the feeding zone (K-W = 851.71, df = 91, after 10,000 permutations, *p* = 1).

**Figure 3:**
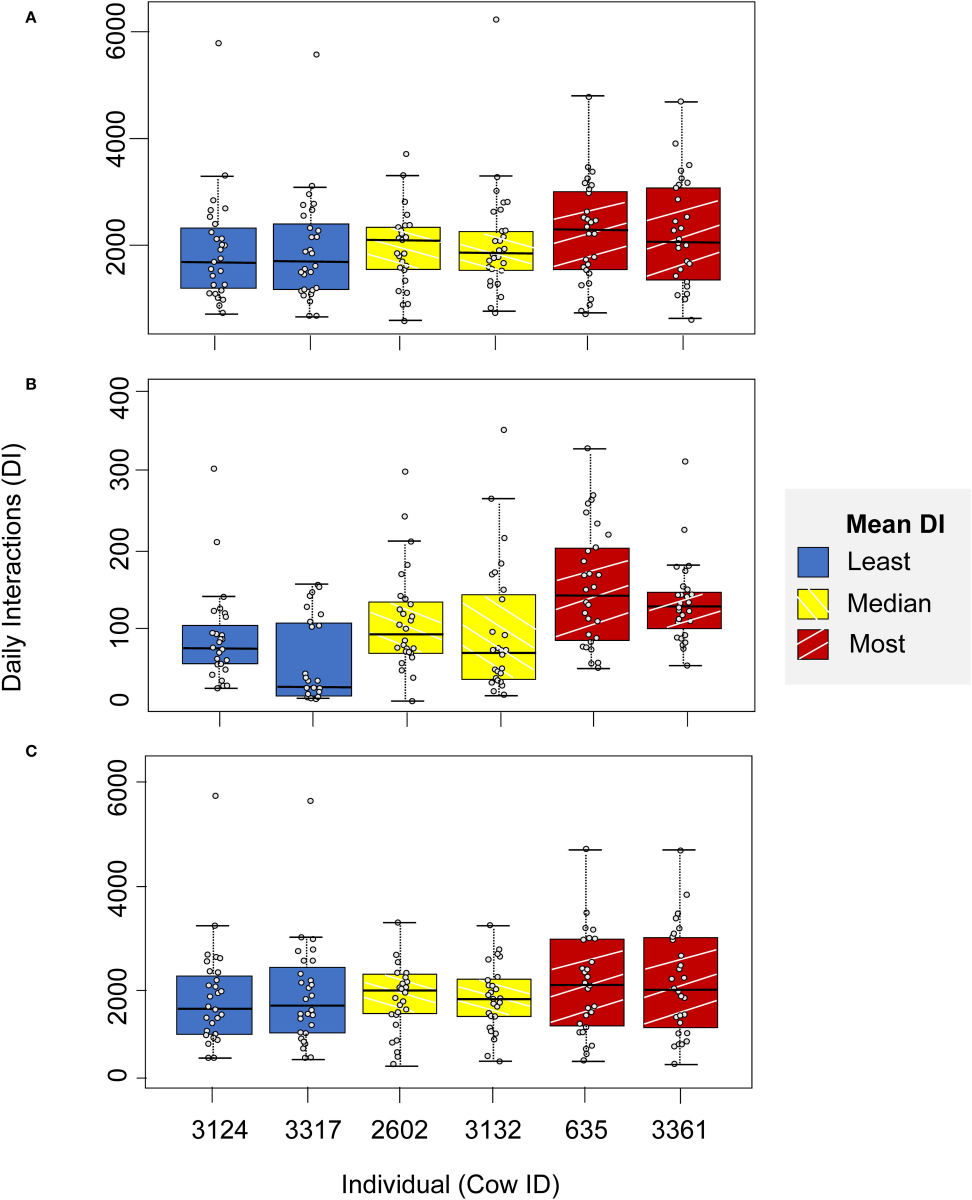
Daily interactions in **(A)** the full barn, **(B)** feeding zone, and **(C)** non-feeding zone, for a highlighted illustrative subset of individuals: two individuals with the least mean daily interactions (blue, cow ID = 3124 and 3317), two with mean daily interactions closest to the median value (yellow, cow ID = 2602 and 3132) and two with the highest mean daily interactions (red, cow ID = 635 and 3361).

Figure 3 illustrates the lack of inter-individual variation in daily interactions in the full barn and the non-feeding zone, and the greater inter-individual variation in daily interactions in the feeding zone for the highlighted subset of individuals.

Social differentiation was observed across the full barn (> 92.96 % of dyads, p < 0.01 across days), the feeding zone (100 % of dyads, *p* < 0.01 across days), and the non-feeding zone (92.96 % of dyads, *p* < 0.01 across days), see Table 1.

### Temporal Variation in Sociality

There was no significant difference in median daily interactions between days in the full barn (K-W chi-squared = 2252.30, df = 27, after 10,000 permutations, *p* = 1; Table 1), feeding zone (K-W = 61.00, df = 27, after 10,000 permutations, *p* = 1; Table 1), nor in the non-feeding zone (K-W = 2268.9, df = 27, respectively after 10,000 permutations, *p* = 1; Table 1).

**Figure 4:**
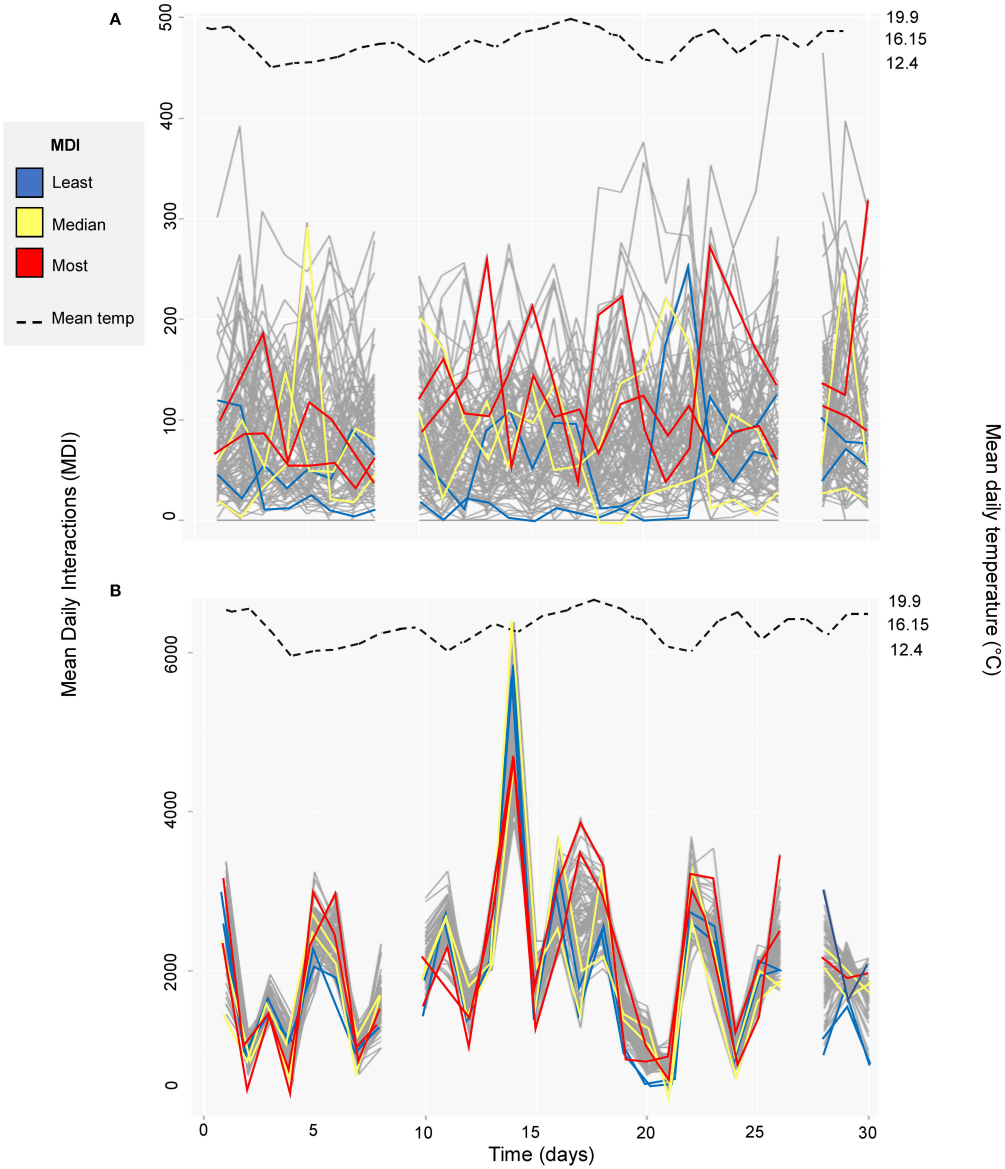
Mean daily interactions across time (01/10/2014 to 31/10/2014 with days excluded from the study omitted) in **(A)** feeding zone, and **(B)** non-feeding zone. An illustrative subset of individuals are highlighted: two individuals with the least mean daily interactions (blue, cow ID = 3124 and 3317), two with mean daily interactions closest to the median value (yellow, cow ID = 2602 and 3132) and two with the highest mean daily interactions (red, cow ID = 635 and 3361). Data for each individual cow is indicated with a gray line. Mean daily temperature is shown with the dashed black line.

Figure 4 highlights the temporal instability in both the functional zone networks. Although there were no clear trends over time, where there were changes these are seen to be highly correlated across all individuals in the feeding zone (*n* = 92; Pearson's coefficient [hereafter ρ] = 0.02, *n* = 92, *p* = 0.90) (Figure 4). Conversely, individual interactions in the feeding zone showed much more random variation than in the non-feeding zone (ρ = 0.55, *n* = 92, *p* < 0.01), as demonstrated with the highlighted subset of individuals (Figure 4). There was a weak but non-significant relationship between mean temperature and mean daily interactions across days in both the feeding zone (ρ = −0.09, df = 26, *p* = 0.66; Table 1; Figure 4) and non-feeding zone (ρ = 0.04, df = 26, *p* = 0.82; Table 1; Figure 4).

**Figure 5:**
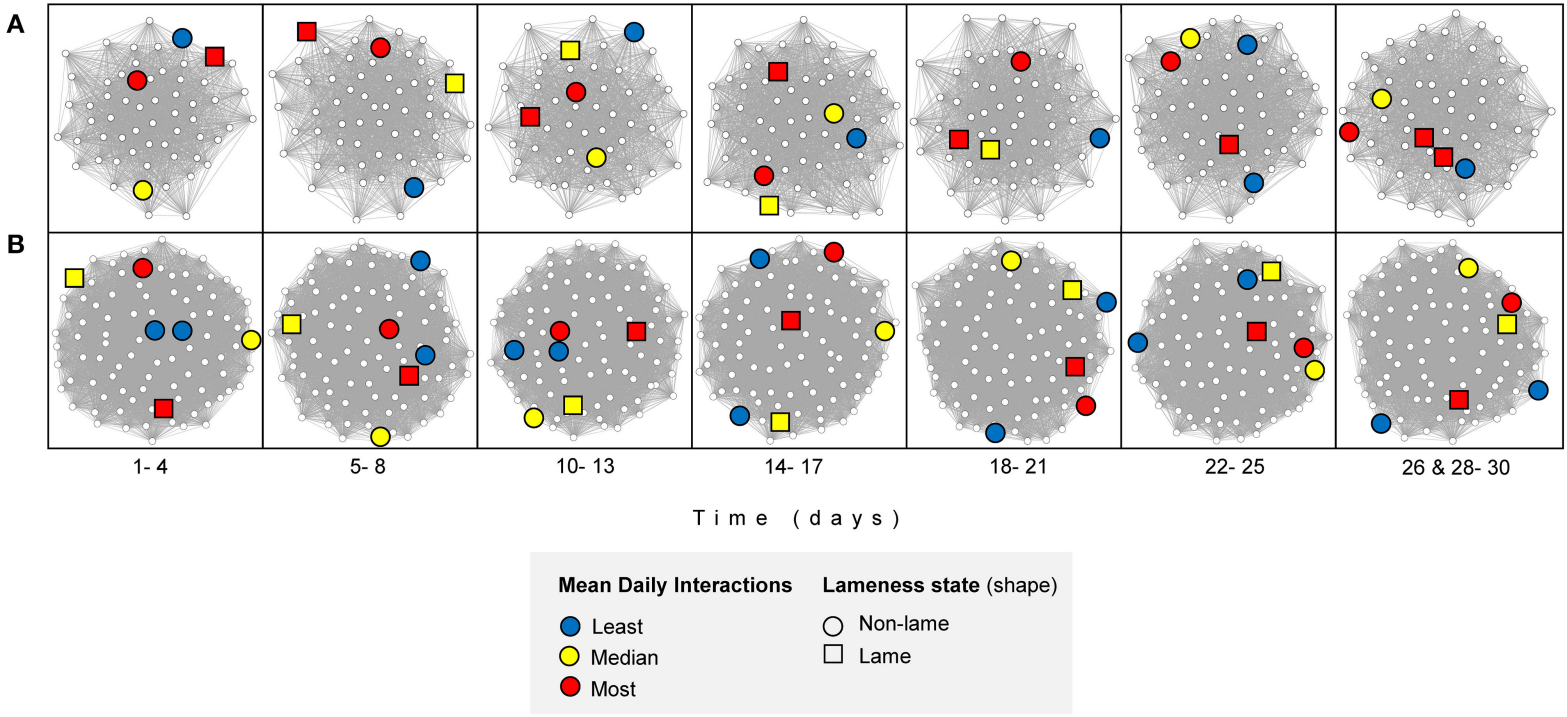
Interaction networks, filtered by mean node degree, over time for **(A)** feeding zone, and **(B)** non-feeding zone. An illustrative subset of individuals are highlighted: two individuals with the least mean daily interactions (blue, cow ID = 3124 and 3317), two with mean daily interactions closest to the median value (yellow, cow ID = 2602 and 3132) and two with the highest mean daily interactions (red, cow ID = 635 and 3361). The Fruchterman-Reingold layout algorithm determined the node positions; unconnected vertices are repelled. Created in RStudio using the “vegan” package (68, 69, 75).

In the feeding zone, there were significant weak positive correlations between all the four-day block averaged- consecutive networks (*n* = 7, comparisons = 6) (range of Spearman's coefficient [hereafter R_s]_ across days = 0.20 to 0.31, after 10,000 permutations and Bonferroni correction, *p* < 0.001 for all comparisons; Table 1; Figure 5). In the full barn there were also weak correlations between the four-day averaged consecutive networks (range of R_s_ = 0.03 to 0.23, after 10,000 permutations and Bonferroni correction, ***p*** **≤** **0.001** for three comparisons (day blocks 1–2, 2–3, 5–6); range of R_s_−0.04 to−0.001, after 10,000 permutations and Bonferroni correction, *p* > 0.23 for three comparisons (day blocks 3–4, 4–5, 6–7). In the non-feeding zone, there were inconsistent weak correlations between consecutive networks (range of R_s_ = 0.05 to 0.24, after 10,000 permutations and Bonferroni correction, ***p*** **<** **0.01** for four comparisons (day blocks 1–2, 2–3, 5–6, 6–7); range of R_s_ = −0.04 to 0.01, after 10,000 permutations and Bonferroni correction, *p* = 1 for two comparisons (day blocks 3–4, 4–5); Table 1; Figure 5). We also conducted this analysis using the original (*n* = 28), and two-, seven- and 14-day blocks, and we obtained qualitatively similar results (Supplementary Material 3 Table 9).

### Impact of Health Status, Parity, and Days in Milk on Sociality

#### Lameness

**Figure 6:**
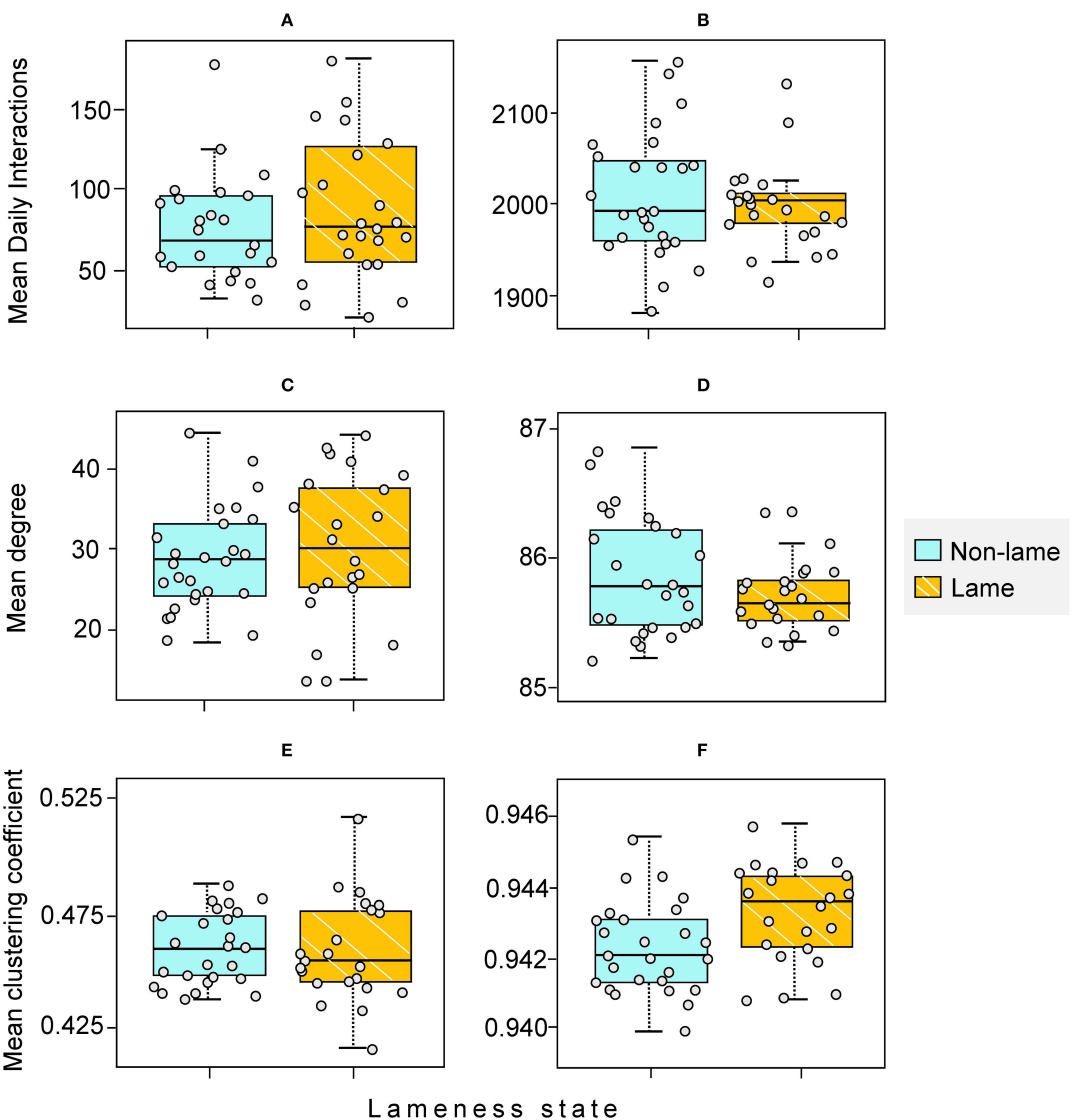
Comparison of Mean Daily Interactions and node-level measures (degree and clustering coefficient) between non-lame (NL) (*n* = 26) and lame (L) (*n* = 22) cows. **(A,C,E)** feeding zone; **(B,D,F)** non-feeding zone. The horizontal line in each boxplot represents the median value. The mean [standard deviation] values for NL and L cows are given, respectively by: **(A)** 74 [35] and 90 [44]; **(B)** 2015 [70] and 2006 [45]; **(C)** 27.92 [8.53] and 30.14 [9.37]; **(D)** 85.85 [0.45] and 85.68 [0.26]; **(E)** 0.44 [0.09] and 0.46 [0.02]; **(F)** 0.94 [0.001] and 0.94 [0.001]. Each individual cow is indicated with a small circle.

Lame cows (*n* = 22) did not have significantly more mean daily interactions than non-lame cows (*n* = 26) in the feeding zone (Wilcoxon test statistic [hereafter W] = 342, *p* = 0.86 after 10,000 permutations; Table 1; Figure 6) nor in the non-feeding zone (W = 276, *p* = 0.40 after 10,000 permutations; Table 1; Figure 6).

In the feeding zone, lame cows did not show a significantly different mean clustering coefficient or degree than non-lame cows (W = 284 and 321.5, respectively, after 10,000 permutations, *p* = 0.53 and 0.25, respectively; Table 1; Figure 6). Similarly, in the non-feeding zone, mean clustering coefficient or degree did not differ between the lameness states (W = 398 and 241.5, after 10,000 permutations, *p* = 0.99 and 0.17 respectively; Table 1; Figure 6).

There was no significant social assortment by lameness in the feeding zone (range of across days R_s_ = −0.06 to 0.04), nor the non-feeding zone (range of across days R_s_ = −0.07 to 0.06) where, after Bonferroni Correction and 10,000 permutations, *p* > 0.80 in all cases for all days (*n* = 28; Table 1). In other words, cows with the same lameness state did not associate more than cows of different lameness states.

#### Parity and Days in Milk

There was no significant social assortment by parity in the feeding zone (range of across days R_s_ = −0.05 to 0.03, after 10,000 permutations and Bonferroni Correction, *p* = 1 for all days; Table 1) or in the non-feeding zone network (range of R_s_ across days = −0.02 to 0.03, after 10,000 permutations and Bonferroni Correction, *p* = 1 for all days; Table 1).

There is also no significant social assortment by DIM in the feeding zone (range of across days R_s_ = −0.03 to 0.04, after 10,000 permutations and Bonferroni Correction *p* = 1 for all days) or the non-feeding zone (range of R_s_ across days = −0.3 to 0.03, after 10,000 permutations and Bonferroni Correction, *p* ≥ 0.44 for all days; Table 1).

The results for social assortment by lameness, parity and DIM in the full barn network were similar to those of the non-feeding zone (results in Table 1).

## Discussion

Within this study we found that the interaction network of the housed dairy herd was highly connected with significant social differentiation, interactions between cows were more heterogenous than expected by chance (18), but the network structure was temporally unstable. There was no evidence of preferential social assortment, showing cows did not associate more than expected by chance according to lameness state, parity, or days in milk (DIM).

Visualization of the full barn interaction network (Figure 2) illustrates that the herd was highly connected, as confirmed by the mean edge density (96%, Table 1). This indicates that each cow was likely to have had interactions with most other cows in the herd each day. It is not clear from this study whether these cows actively seek out and connect with their conspecifics, perhaps to maintain social structure in the group, or whether this high connectivity is a function of the building layout and high stocking density. It must be acknowledged that, due to building works on the farm, the stocking rates were high during our study period (feed space = 0.48 m per cow, lying space = 0.72 cubicles per cow). This may have reduced the ability of the cows to actively choose with whom to be in close proximity with. In agreement with this study, high connectivity was also reported for cows housed in loose straw yards with concrete loafing areas with moderate (to high) stocking rates of 9.50 m^2^ per cow to (7.66 m^2^ per cow) from sensor derived proximity measurements (14, 15). Lower edge densities have been reported in a grazing system, in (7), but in their study an interaction was based on the occurrence of specific behaviors considered to increase the risk of disease transmission rather than social proximity. Lower edge density and a sparse structure was also reported for cows housed in cubicles at a moderate stocking rate (1.03 cubicles per cow), but the group in their study only comprised of 36 cows and interactions were only recorded during two 15 min time slots per day, therefore not capturing changes in location and near neighbors throughout the day (77). Further investigations of dairy cows in a range of housing types and stocking rates are needed to determine if cows are naturally highly connected or whether aspects of the commercial dairy lead to cows spending time in proximity to a greater number of conspecifics.

Analysis on the interaction networks revealed significant inter-individual variation in daily interactions in the non-feeding zone, but not across the feeding zone or when considering the full barn (Figure 3; Table 1). The feeding zone is likely to be a more dynamic location than the loafing and resting areas. Feed bouts are shorter than lying bouts and cows will begin and end their eating bouts at different times, leading to a greater turnaround of contacts at the feed face than other areas of the barn. It is possible however, that cows have greater control over the individual interactions they have in the non-feeding zone and therefore we are able to observe a greater degree of individuality. Researchers have demonstrated that inter-individual variation in sociality is an individual trait in dairy cows (28) influenced by dominance status and personality traits. This may affect an individual's ability to gain resources, such as cubicles, impacting their proximity interactions in the non-feeding zone (17, 21), as also speculated by (14), although we cannot distinguish between these potential factors in this study.

The structure of the interaction network was weakly correlated over time (Figures 3, 4), and individuals periodically isolated from the main network component of the feeding zone (Table 1). These individuals were not the same each day, and they were not of the same lameness status, suggesting their isolation was due to them choosing not to feed at the same time, or being unable to compete due to the lack of space. The overall herd was subject to changes throughout the study period, with the addition and removal of cows outside of the study group (*n* = 92, whole herd = 100–111 cows on a given day), which could have affected the social structure of the herd. In (28), while introductions of new cows to a stable group did not affect the sociality of individual cows, it did weaken the overall social network. The highly connected network in (14) was also subject to changing group composition and the researchers similarly reported weak to moderate correlations in structure between consecutive one-week networks. Further analysis on the temporal stability of dairy cow networks whilst removing specific individuals could aid management.

There were no significant correlations between daily interactions and temperature in this study (Figure 4, Table 1). However, the study period was selected based on there being a relatively stable temperature throughout with temperature low enough not to induce heat stress. Cows have been shown to modify their collective behavior, in terms of clustering for example, or individual behaviors in extreme heat conditions, or show long-term signs of heat stress due to high stocking densities (78–81). Therefore, environmental temperature and even individual cow temperature should be considered when monitoring the herd social structure over longer study periods. Furthermore, the social network may have been more dynamic than initially envisioned due to factors not accounted for, such as farm management actions or treatment interventions (82).

Considering the known social withdrawal response of unhealthy cows (83), it might be predicted that lame cows would be less willing to compete for preferred food or access to cubicles, but no differences in the sociality or positioning were found between lame and non-lame cows (Figure 6, Table 1). At a particularly high stocking rate in intensive cubicle housing, there may have been little opportunity for the 22 “lame” cows identified in this study to self-isolate. Lame cows have been shown to modify their space-use in this barn, but this was with access to an additional loafing area at the end of the cubicle shed which would make social distancing easier than in this study (57). Furthermore, (84) found that lame cows received approximately twice as much allogrooming as cows that were non-lame, and this explanation would also support our finding of no individual-level social assortment by lameness state i.e., cows of the same lameness state did not associate more or less than expected (84) (Table 1). When interpreting the result above we should consider that use of a visual locomotion score is not without the potential for classification errors, especially when scoring large groups of cows at the parlor exit as was the case in this study. It has been reported that mild claw lesions are not always accompanied with a corresponding increase in locomotion score, indicating that locomotion scoring even by trained observers may not be sensitive enough to detect all lameness cases (35). Indeed in a previous study a predictive statistical model correctly classified two cows that were incorrectly classified by observer locomotion scoring (57). Cows with dominant lameness status were also discretely grouped as either “non-lame” or “lame” during analysis (38, 85) (Supplementary Material 1), and these cows may have behaved differently during various time periods of the study. Nonetheless, this study demonstrates a potential way to assess the influence of health status on social interactions within a typical herd. Quantitative measures of individual social interactions and network position may be useful indicators to use within automated monitoring approaches in PLF.

Social differentiation was present in both functional zones (Table 1); some dyads interacted more than others, as similarly shown in (15, 86). A number of previous studies have indicated social differentiation can occur with age, as cows of a similar age would have had greater opportunity to develop social ties with one another (86, 87), particularly if they calved at similar times. In addition, stronger bonds may also form between calves born at similar times, who remain together throughout rearing before joining the milking herd; cows have been shown to invest more time and energy into relationships with herd members sharing long-term experiences (88). Our study does not find that cows differentiate by parity, a proxy for age. While parity may give an indication as to a cow's experience in the herd and may contribute to her personality traits, this measure is probably too coarse to identify cows with historical associations, such as shared calf cohorts, which has been suggested to result in stronger bonds. In this study a recent shared transition period, as indicated by similar DIM, was not sufficient to result in differentiation on this basis. This is in line with the findings of (89), where recent familiarity with cows had no effect on lying down behaviors of cows transitioning to the herd but early familiarity lead to greater synchrony of lying behaviors. Greater detail of the cohorts of cows kept from birth through to the milking herd, unmeasured in this study, may explain the social differentiation observed. It is possible that the high temporal variation of the network structure, and insufficient space within the barn may have impeded the ability to identify these structures. Alternatively, non-random associations may have been the result of cows of similar dominance rank positioning closely, with subordinates displaced from favorable feeding positions by dominant cows (20), particularly as feed space was limited to < 0.60 m/cow. Interactions may be more likely to develop between cows with similar energy requirements and motivation, and hence similar activity time budgets. For example, cows that spend more time eating may spend a lot of time near the feed face and hence position closely to similar cows (15, 86, 87). Stage of lactation affect the time an individual allocates to feeding, given that energy requirements vary with milk yield; for instance, dry matter intake is typically highest during mid-lactation (90).

When interpreting our results, it is important to consider potential limitations of the relatively novel technology and SNA techniques used in this study (82, 91). Although the proximity used to define an interaction was also tested for other radii and time durations, and similar qualitative results were obtained (Supplementary Material 3), any interactions detected were limited by the accuracy of the LPS system (2.66 m mean error for a static sensor). Additionally, a fundamental problem with this type of automated approach to identify proximity interactions is that we are unable to distinguish between which proximity interactions were true social interactions (e.g., allogrooming) and which were non-deliberate or non-social proximity events [e.g., due to the positioning of neighboring cows at the feed face (3, 82) or in cubicles (3)]. Our results are likely to contain both genuine sustained social interactions, as well as proximity events which were not directly social. Distinguishing between genuine social interactions and indirect or non-social proximity interactions is an open research question that requires further investigation. Our chosen proximity identification protocol was tested and validated using observational data and was found to have a sensitivity of 83% (*r* = 3 m and *t* = 60 s), but we were unable to directly estimate the rate of false positives and hence the specificity (Supplementary Material 3). Using a time duration of 60 s is likely to reduce the rate of false positives (compared to using a shorter time duration) but will also potentially exclude genuine social interactions of short duration. Multiple shorter interactions may be as socially relevant as longer sustained interactions. Our analysis was based on a comparison of daily-level network statistics and comparing these over time or between individuals with different lameness status, parity and DIM. It is quite plausible that, although the daily level behavior may be similar across the network, there could be significant individual variability in social interactions on a finer timescale (e.g., hourly or less), particularly around key events such as feeding and milking, and this variability in social behavior may be linked to social status or health. A further limitation is that, although we included the vast majority of cows that were present in the herd throughout the study period (*n* = 92), there were cows that entered and left the group throughout this period, and hence some potential interactions involving these cows would not have been recorded. The effect of missing individuals on the conclusions drawn from a social network analysis are not well understood and this remains an open research question (82, 91). Despite the drawbacks to using proximity to detect potential social interactions, our approach based on using a local positioning system is useful for quickly accumulating the large datasets needed for SNA in an automated way (82).

## Conclusion

A local positioning sensor network was used to automatically monitor the spatial position of a large herd group of permanently housed dairy cows at high temporal resolution for a full month. Proximity interactions were identified by sustained periods of closeness between dyads. The proximity interaction network structure of the herd was highly connected, with significant differentiation in interactions between dyads, and high temporal variability. Lameness, parity, and days in milk were not found to directly influence social interactions or network position. This study demonstrates how automated sensor technology could be used to monitor the social structure of a large commercially relevant group of livestock, and how individual differences in social interactions and network measures could be used to potentially identify health differences between animals. Future work should aim to better distinguish social interactions from indirect non-social interactions and consider how interactions within a larger group may differ in different housing environments and at different stocking densities.

## Data Availability Statement

The raw data supporting the conclusions of this article will be made available by the authors, without undue reservation.

## Ethics Statement

The animal study was reviewed and approved by the Royal Veterinary College Ethics and Welfare Committee under the unique reference number 2012 1223. Written informed consent was obtained from the owners for the participation of their animals in this study.

## Author Contributions

EC, JA, DC, and NB contributed to the study design and secured grant funding. ZB and HH contributed to the study design and undertook data collection with assistance from NB. KC, JV, and EC undertook data analysis with assistance from DC. KC, TC, and EC prepared the manuscript. All authors reviewed and approved the final manuscript.

## Conflict of Interest

NB is employed by company Bos International Ltd. The remaining authors declare that the research was conducted in the absence of any commercial or financial relationships that could be construed as a potential conflict of interest.

## Supplementary Material

Data Sheet 1 Classification of lameness and additional related results. Further details of how individual mobility scores were used to classify lameness states: dominant lame (DL; *n* = 10), lame (L; *n* = 12), dominant non-lame (DNL; *n* = 11) and non-lame (NL; *n* = 15). Additional results are included where the former two groups were each compared to the latter two groups, in terms of mean daily interactions, node degree and clustering coefficient.

Data Sheet 2 Data selection, cleaning, and processing. Further details and justification of the steps taken to select, clean and process the raw positional data collected during the study period. Only cows present throughout the entire duration of the study period were included (*n* = 92), resulting in 21,789,742 location data points. The data cleaning and processing steps resulted in total data removal of‘26%.

Data Sheet 3 Validation of proximity identification protocol and additional results for different time durations and spatial thresholds, and temporal segmentation. We test and validate our algorithm for identifying and classifying proximity interactions against observed proximity events across a range of parameters (spatial threshold radii, *r* = 1–5 m; time duration, *t* = 20-160 s). We include additional results similar to the main paper for these additional parameter values, as well as alternative formats of temporal segmentation of the 28-day study period. In all cases, the results are qualitatively similar to the results given in the main paper and our conclusions hold.

## References

[1] Keeling L Gonyou HW Social Behaviour in Farm Animals. Wallingford, CT: CABI (2001).

[2] Hasegawa N Nishiwaki A Sugawara K Ito I The effects of social exchange between two groups of lactating primiparous heifers on milk production, dominance order, behavior, and adrenocortical response. Appl Anim Behav Sci. (1997) 51:15–27. 10.1016/S0168-1591(96)01082-9

[3] Phillips CJC Rind MI. The effects on production and behavior of mixing uniparous and multiparous cows. J Dairy Sci. (2001) 84:2424–9. 10.3168/jds.S0022-0302(01)74692-9 11768083

[4] Schirmann K Chapinal N Weary DM Heuwieser W von Keyserlingk MAG. Short-term effects of regrouping on behavior of prepartum dairy cows. J Dairy Sci. (2011) 94:2312–9. 10.3168/jds.2010-3639 21524520

[5] von Keyserlingk MAG Olenick D Weary DM. Acute behavioral effects of regrouping dairy cows. J Dairy Sci. (2008) 91:1011–6. 10.3168/jds.2007-0532 18292257

[6] Wey T Blumstein DT Shen W Jordán F Social network analysis of animal behaviour: a promising tool for the study of sociality. Anim Behav. (2008) 75:333–44. 10.1016/j.anbehav.2007.06.020

[7] de Freslon I Martínez-López B Belkhiria J Strappini A Monti G Use of social network analysis to improve the understanding of social behaviour in dairy cattle and its impact on disease transmission. Appl Anim Behav Sci. (2019) 213:47–54. 10.1016/j.applanim.2019.01.006

[8] Croft DP James R Krause J Exploring Animal Social Networks. New Jersey, NJ: Princeton University Press (2008).

[9] Kim J Hastak M Social network analysis: characteristics of online social networks after a disaster. Int J Inf Manag. (2018) 38:86–96. 10.1016/j.ijinfomgt.2017.08.003

[10] Otte E Rousseau R Social network analysis: a powerful strategy, also for the information sciences. J Inf Sci. (2002) 28:441–53. 10.1177/016555150202800601

[11] CHOI J Barnett G CHON B-S Comparing World City Networks: a network analysis of Internet backbone and air transport intercity linkages. Glob Netw. (2006) 6:81–99. 10.1111/j.1471-0374.2006.00134.x

[12] Makagon MM McCowan B Mench JA. How can social network analysis contribute to social behavior research in applied ethology? Appl Anim Behav Sci. (2012) 138:3. 10.1016/j.applanim.2012.02.003 24357888 PMC3865988

[13] Croft D Darden S Wey T Current directions in animal social networks. Curr Opin Behav Sci. (2016) 12:52–8. 10.1016/j.cobeha.2016.09.001

[14] Boyland NK Mlynski DT James R Brent LJN Croft DP The social network structure of a dynamic group of dairy cows: from individual to group level patterns. Appl Anim Behav Sci. (2016) 174:1–10. 10.1016/j.applanim.2015.11.016

[15] Gygax L Neisen G Wechsler B Socio-spatial relationships in dairy cows. Ethology. (2010) 116:10–23. 10.1111/j.1439-0310.2009.01708.x

[16] Hodges HR An Investigation of Social Structure in Housed Dairy Cows. (2018) Available online at: http://repository.essex.ac.uk/23324/ (accessed July 11, 2020).

[17] Neave HW Costa JHC Weary DM von Keyserlingk MAG. Long-term consistency of personality traits of cattle. R Soc Open Sci. (2020) 7:191849. 10.1098/rsos.191849 32257341 PMC7062087

[18] Boyland NK The Influence of Social Networks on Welfare and Productivity in Dairy Cattle. (2015) Available online at: https://ore.exeter.ac.uk/repository/handle/10871/19360 (accessed July 11, 2020).

[19] Rioja-Lang FC Roberts DJ Healy SD Lawrence AB Haskell MJ. Dairy cows trade-off feed quality with proximity to a dominant individual in Y-maze choice tests. Appl Anim Behav Sci. (2009) 117:159–64. 10.1016/j.applanim.2008.12.00322720949

[20] Val-Laillet D Rushen J Keyserlingk M The concept of social dominance and the social distribution of feeding-related displacements between cows. Appl Anim Behav Sci. (2007) 111:158–72. 10.1016/j.applanim.2007.06.001

[21] de Freslon I Peralta JM Strappini AC Monti G. Understanding allogrooming through a dynamic social network approach: an example in a group of dairy cows. Front Vet Sci. (2020) 7:535. 10.3389/fvets.2020.00535 32851054 PMC7417353

[22] Foris B Zebunke M Langbein J Melzer N Comprehensive analysis of affiliative and agonistic social networks in lactating dairy cattle groups. Appl Anim Behav Sci. (2019) 210:60–7. 10.1016/j.applanim.2018.10.016

[23] Schieltz JM Okanga S Allan BF Rubenstein DI GPS tracking cattle as a monitoring tool for conservation and management. Afr J Range Forage Sci. (2017) 34:173–7. 10.2989/10220119.2017.1387175

[24] Duncan S Stewart TI Oliver M Mavoa S MacRae D Badland HM. Portable global positioning system receivers: static validity and environmental conditions. Am J Prev Med. (2013) 44:e19–e29. 10.1016/j.amepre.2012.10.013 23332343

[25] Rice EM Ferrell J Vanzant E Jackson J Costa J Real-time localization system for livestock dairy cattle: validation of static positioning in a commercial facility. In: ASABE ASABE Annual International Virtual Meeting. St. Joseph, MI (2020). 10.13031/aim.202000797

[26] Gygax L Neisen G Bollhalder H Accuracy, and validation of a radar-based automatic local position measurement system for tracking dairy cows in free-stall barns. Comput Electron Agric. (2007) 56:23–33. 10.1016/j.compag.2006.12.004

[27] Tullo E Fontana I Gottardo D Sloth KH Guarino M. Technical note: validation of a commercial system for the continuous and automated monitoring of dairy cow activity. J Dairy Sci. (2016) 99:7489–94. 10.3168/jds.2016-11014 27344390

[28] Rocha LEC Terenius O Veissier I Meunier B Nielsen PP Persistence of sociality in group dynamics of dairy cattle. Appl Anim Behav Sci. (2020) 223:104921 10.1016/j.applanim.2019.104921

[29] Whitehead H Analyzing Animal Societies: Quantitative Methods for Vertebrate Social Analysis. Chicago, IL: University of Chicago Press (2008).

[30] Ballerini M Cabibbo N Candelier R Cavagna A Cisbani E Giardina I. Interaction ruling animal collective behavior depends on topological rather than metric distance: evidence from a field study. Proc Natl Acad Sci. (2008) 105:1232–7. 10.1073/pnas.0711437105 18227508 PMC2234121

[31] Nagy M Vásárhelyi G Pettit B Roberts-Mariani I Vicsek T Biro D. Context-dependent hierarchies in pigeons Máté Nagy. Proc Natl Acad Sci USA. (2013) 110:13049–54. 10.1073/pnas.1305552110 23878247 PMC3740899

[32] Griffiths BE Grove White D Oikonomou G. A cross-sectional study into the prevalence of dairy cattle lameness and associated herd-level risk factors in england and wales. Front Vet Sci. (2018) 5:65. 10.3389/fvets.2018.00065 29675419 PMC5895762

[33] Kofler J Pesenhofer R Landl G Sommerfeld-Stur I Peham C. Monitoring of dairy cow claw health status in 15 herds using the computerised documentation program Claw Manager and digital parameters. Tierarztl Prax Ausg G Grosstiere Nutztiere. (2013) 41:31–44. 10.1055/s-0038-1623146 23403757

[34] Thompson AJ Weary DM Bran JA Daros RR Hötzel MJ von Keyserlingk MAG. Lameness and lying behavior in grazing dairy cows. J Dairy Sci. (2019) 102:6373–82. 10.3168/jds.2018-15717 31079902

[35] Tadich N Flor E Green L. Associations between hoof lesions and locomotion score in 1098 unsound dairy cows. Vet J. (2010) 184:60–5. 10.1016/j.tvjl.2009.01.005 19211281

[36] von Keyserlingk MAG Barrientos A Ito K Galo E Weary DM. Benchmarking cow comfort on North American freestall dairies: lameness, leg injuries, lying time, facility design, and management for high-producing Holstein dairy cows. J Dairy Sci. (2012) 95:7399–408. 10.3168/jds.2012-5807 23063152

[37] Amory JR Barker ZE Wright JL Mason SA Blowey RW Green LE. Associations between sole ulcer, white line disease and digital dermatitis and the milk yield of 1824 dairy cows on 30 dairy cow farms in England and Wales from February 2003–November 2004. Prev Vet Med. (2008) 83:381–91. 10.1016/j.prevetmed.2007.09.007 18031851

[38] Archer SC Green MJ Huxley JN. Association between milk yield and serial locomotion score assessments in UK dairy cows. J Dairy Sci. (2010) 93:4045–53. 10.3168/jds.2010-3062 20723678

[39] Barker ZE Amory JR Wright JL Mason SA Blowey RW Green LE. Risk factors for increased rates of sole ulcers, white line disease, and digital dermatitis in dairy cattle from twenty-seven farms in England and Wales. J Dairy Sci. (2009) 92:1971–8. 10.3168/jds.2008-1590 19389954

[40] Hernandez-Mendo O von Keyserlingk MAG Veira DM Weary DM. Effects of pasture on lameness in dairy cows. J Dairy Sci. (2007) 90:1209–14. 10.3168/jds.S0022-0302(07)71608-9 17297096

[41] Thomas AP Dipu MT Lameness in dairy cattle: nutritional approaches for prevention and management. Indian Vet J. (2014) 12:18–22. Available online at: http://jivaonline.net/archive/download.php?file=pdf_222.pdf&id=222

[42] Miguel-Pacheco GG Thomas HJ Huxley JN Newsome RF Kaler J. Effect of claw horn lesion type and severity at the time of treatment on outcome of lameness in dairy cows. Vet J. (2017) 225:16–22. 10.1016/j.tvjl.2017.04.015 28720293

[43] Leach KA Tisdall DA Bell NJ Main DCJ Green LE. The effects of early treatment for hindlimb lameness in dairy cows on four commercial UK farms. Vet J. (2012) 193:626–32. 10.1016/j.tvjl.2012.06.043 22884565

[44] Randall LV Green MJ Chagunda MGG Mason C Green LE Huxley JN. Lameness in dairy heifers; impacts of hoof lesions present around first calving on future lameness, milk yield and culling risk. Prev Vet Med. (2016) 133:52–63. 10.1016/j.prevetmed.2016.09.006 27720027 PMC5063951

[45] Newsome RF Green MJ Bell NJ Bollard NJ Mason CS Whay HR. A prospective cohort study of digital cushion and corium thickness. Part 2: Does thinning of the digital cushion and corium lead to lameness and claw horn disruption lesions? J Dairy Sci. (2017) 100:4759–71. 10.3168/jds.2016-12013 28434731

[46] Tunstall J Mueller K Grove White D Oultram JWH Higgins HM. Lameness in beef cattle: UK farmers' perceptions, knowledge, barriers, and approaches to treatment and control. Front Vet Sci. (2019) 6:94. 10.3389/fvets.2019.00094 30984772 PMC6449762

[47] Leach KA Whay HR Maggs CM Barker ZE Paul ES Bell AK. Working towards a reduction in cattle lameness: 1. Understanding barriers to lameness control on dairy farms. Res Vet Sci. (2010) 89:311–7. 10.1016/j.rvsc.2010.02.014 20363487

[48] Bran JA Daros RR von Keyserlingk MAG Hötzel MJ. Lameness on Brazilian pasture based dairies—part 1: Farmers' awareness and actions. Prev Vet Med. (2018) 157:134–41. 10.1016/j.prevetmed.2018.06.007 30086841

[49] Dahl-Pedersen K Foldager L Herskin MS Houe H Thomsen PT. Lameness scoring and assessment of fitness for transport in dairy cows: agreement among and between farmers, veterinarians, and livestock drivers. Res Vet Sci. (2018) 119:162–6. 10.1016/j.rvsc.2018.06.017 29940460

[50] Weigele HC Gygax L Steiner A Wechsler B Burla J-B. Moderate lameness leads to marked behavioral changes in dairy cows. J Dairy Sci. (2018) 101:2370–82. 10.3168/jds.2017-13120 29290435

[51] Dairy AHDB. Available online at: https://ahdb.org.uk/dairy#.Xv4SPihKg2w (accessed July 15, 2020).

[52] Tadich N Tejeda C Bastias S Rosenfeld C Green LE. Nociceptive threshold, blood constituents and physiological values in 213 cows with locomotion scores ranging from normal to severely lame. Vet J Lond Engl. (2013) 197:401–5. 10.1016/j.tvjl.2013.01.029 23499542

[53] Barker ZE Vázquez Diosdado JA Codling EA Bell NJ Hodges HR Croft DP. Use of novel sensors combining local positioning and acceleration to measure feeding behavior differences associated with lameness in dairy cattle. J Dairy Sci. (2018) 101:6310–21. 10.3168/jds.2016-12172 29705427

[54] Palmer MA Law R O'Connell NE Relationships between lameness and feeding behaviour in cubicle-housed Holstein–Friesian dairy cows. Appl Anim Behav Sci. (2012) 140:121–7. 10.1016/j.applanim.2012.06.005

[55] Blackie N Bleach E Amory J Scaife J Impact of lameness on gait characteristics and lying behaviour of zero grazed dairy cattle in early lactation. Appl Anim Behav Sci. (2011) 129:67–73. 10.1016/j.applanim.2010.10.006

[56] Alsaaod M Römer C Kleinmanns J Hendriksen K Rose-Meierhöfer S Plümer L Electronic detection of lameness in dairy cows through measuring pedometric activity and lying behavior. Appl Anim Behav Sci. (2012) 142:134–41. 10.1016/j.applanim.2012.10.001

[57] Vázquez Diosdado JA Barker ZE Hodges HR Amory JR Croft DP Bell NJ. Space-use patterns highlight behavioural differences linked to lameness, parity, and days in milk in barn-housed dairy cows. PLoS ONE. (2018) 13:e208424. 10.1371/journal.pone.0208424 30566490 PMC6300209

[58] Cramer MC Stanton AL. Associations between health status and the probability of approaching a novel object or stationary human in preweaned group-housed dairy calves. J Dairy Sci. (2015) 98:7298–308. 10.3168/jds.2015-9534 26254525

[59] Cramer MC Ollivett TL Stanton AL. Associations of behavior-based measurements and clinical disease in preweaned, group-housed dairy calves. J Dairy Sci. (2016) 99:7434–43. 10.3168/jds.2015-10207 27372593

[60] Fogsgaard KK Røntved CM Sørensen P Herskin MS. Sickness behavior in dairy cows during *Escherichia coli* mastitis. J Dairy Sci. (2012) 95:630–8. 10.3168/jds.2011-4350 22281328

[61] Dittrich I Gertz M Krieter J. Alterations in sick dairy cows' daily behavioural patterns. Heliyon. (2019) 5:e02902. 10.1016/j.heliyon.2019.e02902 31799469 PMC6881618

[62] Belaid MA Rodríguez-Prado M Rodríguez-Prado DV Chevaux E Calsamiglia S. Using behavior as an early predictor of sickness in veal calves. J Dairy Sci. (2020) 103:1874–83. 10.3168/jds.2019-16887 31521341

[63] Goldhawk C Chapinal N Veira DM Weary DM von Keyserlingk MAG. Prepartum feeding behavior is an early indicator of subclinical ketosis. J Dairy Sci. (2009) 92:4971–7. 10.3168/jds.2009-2242 19762814

[64] Patbandha TK Mohanty TK Layek SS Kumaresan A Behera K Application of pre-partum feeding and social behaviour in predicting risk of developing metritis in crossbred cows. Appl Anim Behav Sci. (2012) 139:10–7. 10.1016/j.applanim.2012.03.014

[65] Sepúlveda-Varas P Proudfoot KL Weary DM von Keyserlingk MAG Changes in behaviour of dairy cows with clinical mastitis. Appl Anim Behav Sci. (2016) 175:8–13. 10.1016/j.applanim.2014.09.022

[66] Galindo F Broom DM. The effects of lameness on social and individual behavior of dairy cows. J Appl Anim Welf Sci. (2002) 5:193–201. 10.1207/S15327604JAWS0503_03 12578740

[67] Overton MW Sischo WM Temple GD Moore DA. Using time-lapse video photography to assess dairy cattle lying behavior in a free-stall barn. J Dairy Sci. (2002) 85:2407–13. 10.3168/jds.S0022-0302(02)74323-3 12362476

[68] R: The R Project for Statistical Computing Available online at: https://www.r-project.org/ (accessed July 11, 2020).

[69] RStudio Team RStudio: Integrated Development for R. RStudio (2020). Available online at: https://rstudio.com/products/rstudio/ (accessed July 11, 2020).

[70] Csardi G Nepusz T igraph: Network Analysis and Visualization. (2020). Available online at: https://CRAN.R-project.org/package=igraph (accessed July 11, 2020).

[71] Farine D. A guide to null models for animal social network analysis. Methods Ecol Evol. (2017) 8:1309–20. 10.1111/2041-210X.12772 29104749 PMC5656331

[72] Farine DR Whitehead H. Constructing, conducting and interpreting animal social network analysis. J Anim Ecol. (2015) 84:12418. 10.1111/1365-2656.12418 26172345 PMC4973823

[73] Phipson B Smyth GK. Permutation p-values should never be zero: calculating exact p-values when permutations are randomly drawn. Stat Appl Genet Mol Biol. (2010) 9:1585. 10.2202/1544-6115.1585 21044043

[74] Mantel N The detection of disease clustering and a generalized regression approach. Cancer Res. (1967) 27:209–20. 6018555

[75] Oksanen J Blanchet FG Friendly M Kindt R Legendre P McGlinn D vegan: Community Ecology Package. (2019). Available online at: https://CRAN.R-project.org/package=vegan (accessed October 30, 2020).

[76] Krause J Croft DP James R. Social network theory in the behavioural sciences: potential applications. Behav Ecol Sociobiol. (2007) 62:15–27. 10.1007/s00265-007-0445-8 32214613 PMC7079911

[77] Salau J Lamp O Krieter J Dairy cows' contact networks derived from videos of eight cameras. Biosyst Eng. (2019) 188:106–13. 10.1016/j.biosystemseng.2019.10.018

[78] Allen JD Hall LW Collier RJ Smith JF. Effect of core body temperature, time of day, and climate conditions on behavioral patterns of lactating dairy cows experiencing mild to moderate heat stress. J Dairy Sci. (2015) 98:118–27. 10.3168/jds.2013-7704 25468707

[79] Herbut P Angrecka S Full article: relationship between THI level and dairy cows' behaviour during summer period. Ital J Anim Sci. (2018) 17:226–33. 10.1080/1828051X.2017.1333892

[80] Shahriar MdS Smith D Rahman A Henry D Bishop-Hurley G Rawnsley R Heat event detection in dairy cows with collar sensors: an unsupervised machine learning approach. In: *2015 IEEE SENSORS* Busan (2015). p. 1–4. 10.1109/ICSENS.2015.7370528

[81] Shahriar MdS Smith D Rahman A Freeman M Hills J Rawnsley R Detecting heat events in dairy cows using accelerometers and unsupervised learning. Comput Electron Agric. (2016) 128:20–6. 10.1016/j.compag.2016.08.009

[82] James R Croft DP Krause J Potential banana skins in animal social network analysis. Behav Ecol Sociobiol. (2009) 63:989–97. 10.1007/s00265-009-0742-5

[83] Dantzer R. Cytokine-induced sickness behavior: where do we stand? Brain Behav Immun. (2001) 15:7–24. 10.1006/brbi.2000.0613 11259077

[84] Galindo F Broom DM. The relationships between social behaviour of dairy cows and the occurrence of lameness in three herds. Res Vet Sci. (2000) 69:75–9. 10.1053/rvsc.2000.0391 10924398

[85] Groenevelt M Main DCJ Tisdall D Knowles TG Bell NJ. Measuring the response to therapeutic foot trimming in dairy cows with fortnightly lameness scoring. Vet J. (2014) 201:283–8. 10.1016/j.tvjl.2014.05.017 24881511

[86] Boyland NK James R Mlynski DT Madden JR Croft DP Spatial proximity loggers for recording animal social networks: consequences of inter-logger variation in performance. Behav Ecol Sociobiol. (2013) 67:1877–90. 10.1007/s00265-013-1622-6

[87] Harris NR Johnson DE McDougald NK George MR Social associations and dominance of individuals in small herds of cattle. Rangel Ecol Manag. (2007) 60:339–49. 10.2111/1551-5028(2007)60[339:SAADOI]2.0.CO;2

[88] Gutmann AK Špinka M Winckler C Long-term familiarity creates preferred social partners in dairy cows. Appl Anim Behav Sci. (2015) 169:1–8. 10.1016/j.applanim.2015.05.007

[89] Gutmann AK Špinka M Winckler C Do familiar group mates facilitate integration into the milking group after calving in dairy cows? Appl Anim Behav Sci. (2020) 229:105033 10.1016/j.applanim.2020.105033

[90] Managing Cow Lactation Cycles Available online at: http://www.thecattlesite.com/articles/4248/managing-cow-lactation-cycles/ (accessed July 14, 2020).

[91] Croft DP Madden JR Franks DW James R. Hypothesis testing in animal social networks. Trends Ecol Evol. (2011) 26:502–7. 10.1016/j.tree.2011.05.012 21715042

