# Supplementary material for: Proximity Interactions in a Permanently Housed Dairy Herd: Network Structure, Consistency, and Individual Differences
Source: Front Vet Sci. 2020 Dec 7;7:583715. doi: 10.3389/fvets.2020.583715 (PMC7750390; doi:10.3389/fvets.2020.583715)
Supplement: Data Sheet 1 — Classification of lameness and additional related results. Further details of how individual mobility scores were used to classify lameness states: dominant lame (DL; n = 10), lame (L; n = 12), dominant non-lame (DNL; n = 11) and non-lame (NL; n = 15). Additional results are included where the former two groups were each compared to the latter two groups, in terms of mean daily interactions, node degree and clustering coefficient. [file Data_Sheet_1.docx]

Supplementary Material 1

# Classifying lameness state

Cows were assigned a mobility score fortnightly (30/9/2014, 13/10/2014, and 27/10/2014) using the AHDB mobility score(1). Amobility score of 0-3 was assigned, where 0 is good mobility, 1 is imperfect mobility, 2 is impaired mobility and 3 is severely impaired mobility. If a score was not recorded, ‘NS’ was noted.All cows in each session were scored by ZB who had scored approximately 70,000 cows over ten years before this study. The average observer reliability, score blind 0- 3 by ZB using AHDB dairy standardized videos (1), was 80 % for two sessions (19/05/2013 (n = 10) and 18/02/2015 (n = 10).

For this study, cows with score 2 or 3 were considered as clinically lame (L) and cows with scores 0 or 1 were considered non-lame (NL). Cows scored as non-lame for two successive scoring sessions (NL-NL-L or L-NL-NL) were classed as ‘dominant not lame’ (DNL), and cows scored as lame for most sessions (L-L-NL or NL-L-L) were classed as ‘dominant lame’ (DL). Cows that apparently changed status twice within the study (NL-L-NL or L-NL-L), or those with missing data (no recordings in one or more of the sessions, typicallydue to the individual not being sighted) were not included in the lameness classification, see Supplementary Material 1 Table 1.

**Supplementary Material 1Table1.**Classified lameness status of (n = 92) dairy cows within the study.

| **Lameness status** | **Frequency (number of cows)** |
| --- | --- |
| Non-lame (NL) | 15 |
| Lame (L) | 12 |
| Dominant non-lame (DNL) | 11 |
| Dominant lame (DL) | 10 |
| Multiple change of status | 14 |
| No/ missing data | 30 |
| Total | 92 |

# Analysis including dominant lame and dominant non-lame classification

# Toanalyse differences between lameness states, we combined NL and DNL cows into ‘non-lame cows’ (n = 26) and then L and DL cows into ‘lame cows’ (n = 22)and report these results in the main paper. Here, we report results within these two groups, comparing the NL and DNL, then the L and DL (using our protocol to define an interaction, as when cows are within a 3m radius for at least 60 s). There were no real significant differences in node degreesandclustering coefficients within the groups.

In the feeding zone, there weresignificant differences in mean daily interactions, between DNL and NL cows (Wilcox test statistic [hereafter W] = 89, after 10000 permutations, p < 0.001), and between DL and L cows (W = 69, after 10,000 permutations p < 0.001), see Supplementary Material 1 Figure 1A. In the non-feeding zone, there were nosignificant differences in mean daily interactions, between DNL and NL cows (W = 89, after 10,000 permutations, p = 0.03) or between DL and L cows (W = 51.5, after 10000 permutations, p = 0.73), see Supplementary Material 1Figure 1B.

In the feeding zone, there were no significant differences in node-level measures (mean degree or mean clustering coefficient) between NL cows and DNL cows (respectively W =101.5, p = 0.20; W =111, p = 0.10; 10,000 permutations; Supplementary Material 1Figure 1C and 1E), or between L and DL cows (respectively W = 59, p =0.56; W =61, p = 0.50; 10,000 permutations; Supplementary Material 1Figure 1C and 1E).

In the non-feeding zone, there were no significant differences in meandegree or clustering coefficient betweenNL and DNL cows (respectively W = 70.5, p = 0.77; W = 69, p = 0.79; 10,000 permutations; Supplementary Figure Material 11D and 1F), or between L and DL cows (respectively W = 62, p = 0.46; W = 68, p = 0.32; 10,000 permutations; Supplementary Material 1Figure 1D and 1F).


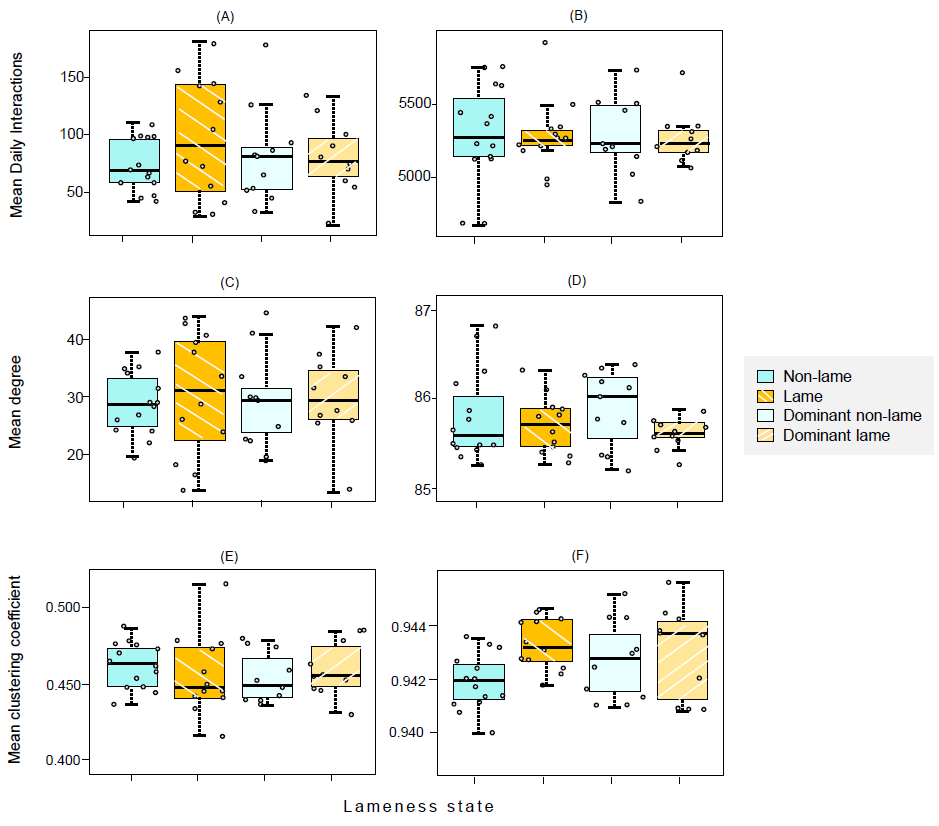


**Supplementary Material 1Figure1.**Mean daily interactions and mean node-level measures (degree and clustering coefficient),calculated across the study period for each individual cow, and compared between non-lame (NL), lame (L), dominant non-lame (DNL), and dominant lame (DL) cows. (A, C, E) feeding zone; (B, D, F) non-feeding zone. Data for each individual cow is indicated with a small circle.

**References**

1. Dairy | AHDB. Available at: https://ahdb.org.uk/dairy#.Xv4SPihKg2w [Accessed July 15, 2020]
