# Supplementary material for: Proximity Interactions in a Permanently Housed Dairy Herd: Network Structure, Consistency, and Individual Differences
Source: Front Vet Sci. 2020 Dec 7;7:583715. doi: 10.3389/fvets.2020.583715 (PMC7750390; doi:10.3389/fvets.2020.583715)
Supplement: Data Sheet 2 — Data selection, cleaning, and processing. Further details and justification of the steps taken to select, clean and process the raw positional data collected during the study period. Only cows present throughout the entire duration of the study period were included (n = 92), resulting in 21,789,742 location data points. The data cleaning and processing steps resulted in total data removal of‘26%. [file Data_Sheet_2.docx]

***Supplementary Material 2***

# Data selection

Due to system malfunction, three days within the study period (09/10/2014, 27/10/2014 and 31/10/2014) were excluded.Within the remaining (d = 28) days of the study period the maximum number of cows in the barn on a given day varied from 100 to 111 (mean =105 cows, standard error = 0.59). Any cows that were only partially present during the study period (i.e. were present for < 28 days) were excluded from the analysis. A total of 92 cows were present entirely throughout the 28-day entire study period and were included in the analysis. From these 92 cows,21,789,742location data points were collected (due to a momentary loss of data, approximately 30 minutes per day [approximately 28 x 30 x 6 x 92], when the system reset around midnight).

# Data cleaning and processing

Data cleaning and processing was completed in three steps, resulting in a total data removal of 26.05%(see flow chart in Supplementary Fig 1).


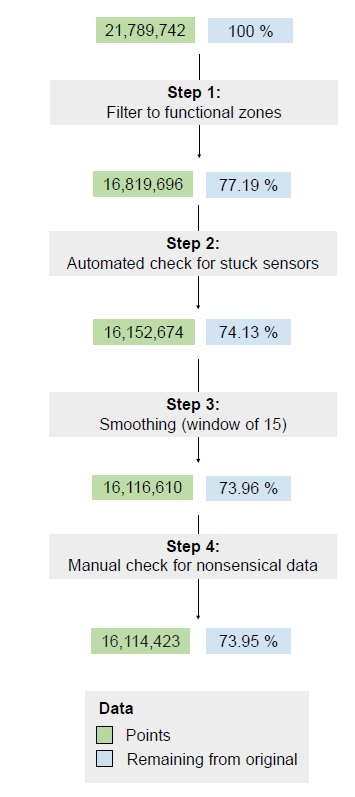


**Supplementary Material 2 Fig 1.** Overview of the data cleaning and processing steps. Step 1: filtering data points to those detected within the functional zones; step 2: automated check for stuck sensors (x and/or y coordinate(s) exactly the same for two or more consecutive time points); step 3: smoothing (simple moving average with a window of 15 time points); step 4: manual check for nonsensical data (cows seemingly restricted to an unrealistically small area for a full day).

*2.1 Filtering to functional zones*

Data recorded outside of the defined feeding and non-feeding functional zones (which included a 3m buffer region to allow for minor positional errors) were excluded from analysis. This included (correct) location data from the milking parlour and collecting yard when cows were constrained during milking periods, as well as erroneous data from non-milking periods where locations were (incorrectly) recorded more than 3m outside the barn area. Of the total data points collected, 30.71% (4,970,046) were located outside of the barn area and were removed in this first step(Step 1, Supplementary Material 2 Fig 1).

*2.2 Automated removal of ‘stuck’ sensor data*

An automated algorithm, created for the purposes of this study, was used to detect datawhere the sensor was located at exactly the same *x*and/or*y* coordinate(s) for two or more consecutive time points (3.06% of data, 667,022points; Step 2 in Supplementary Fig 1). This happened occasionally when a sensor was lost from the system; in such cases its last known location is continually reported, leading to an apparently ‘stuck’ sensor at exactly the same location over time. Additionally, given the typical known noise and error within the sensor system, and the natural movement of the cows, we can be certain that a properly functioning sensor would never report the exact same location coordinates over successive time points if it is working correctly (even sensors at rest and not in use would still report very minor variations in location). Supplementary Material 2 Figure 2 shows an illustrative example of instances when sensors were working (A-B) and ‘stuck’ at both *x* and *y*coordinates (C-D).


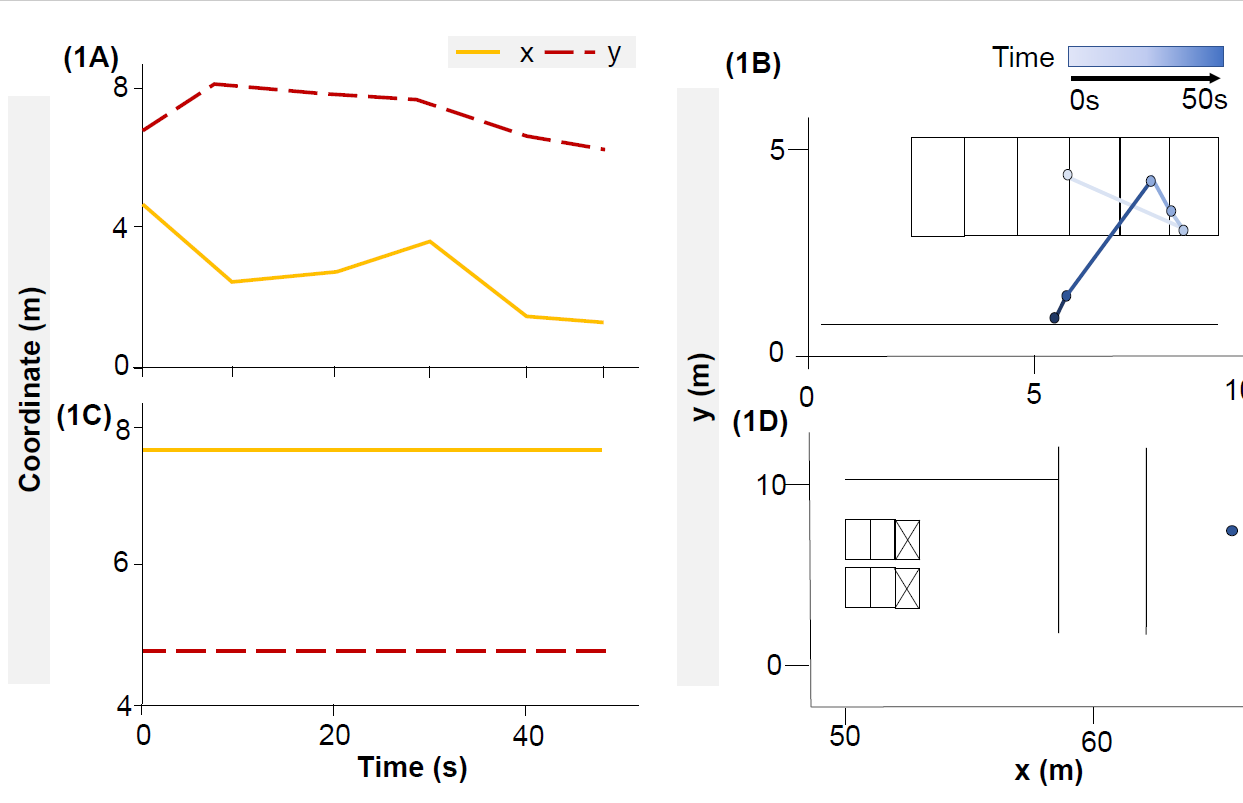


**Supplementary Fig 2.**(A) Coordinates of sensor 0002f1 (cow 2352) on 08/10/2014 from 05:39:53 (time = 0s) to 05:40:43 (time = 50s); (B) the same coordinates plotted as a trajectory in a zoomed-in region of the barn (see Fig 1 in main paper). (C,D) Coordinates and trajectory (single location point) of sensor 0000ee (cow 3233) for the same day and time period, illustrating how this sensor was apparently ‘stuck’ and was recording incorrect location data.

*2.3 Smoothing*

The remaining data were smoothed using a simple moving average (SMA, see Equation S1 below) with a two-sided window of 15 data points to smooth location outliers and to reduce instantaneous noise:

$$SMA_{15} = \frac{{(x, y)}_{t-7}+ {(x, y)}_{t-1}+\ldots{(x, y)}_{t}{+\ldots(x, y)}_{t+1}+ {(x, y)}_{t+7}}{15}\mathbf{(S1)}$$

where(*x, y)* = position and*t* = time. To apply the SMA with a window size of 15 time points, it is necessary to remove seven data points from the start and end of the location time series for each sensor per day. In total this led to 0.17 % of the data being removed(36,064 = 14 data points x 92 cows x 28 days; Step 3 in Supplementary Fig 1).

Supplementary Material 2 Figure 3A shows an illustrative example of samples of the raw trajectories of three cows (1317, 2352 and 88) over a 5-minute period on 1/10/2014. Prior to smoothing, cow 1317 was detected to have interacted with cow 88 during this time,using a proximity threshold of 3 m, whereas after smoothingthe cowwas not detected tohave interacted with any other cows during this time. Cows 2352 and 88 were detected to have interactedboth before and after smoothing(Supplementary Material 2Fig 3B).


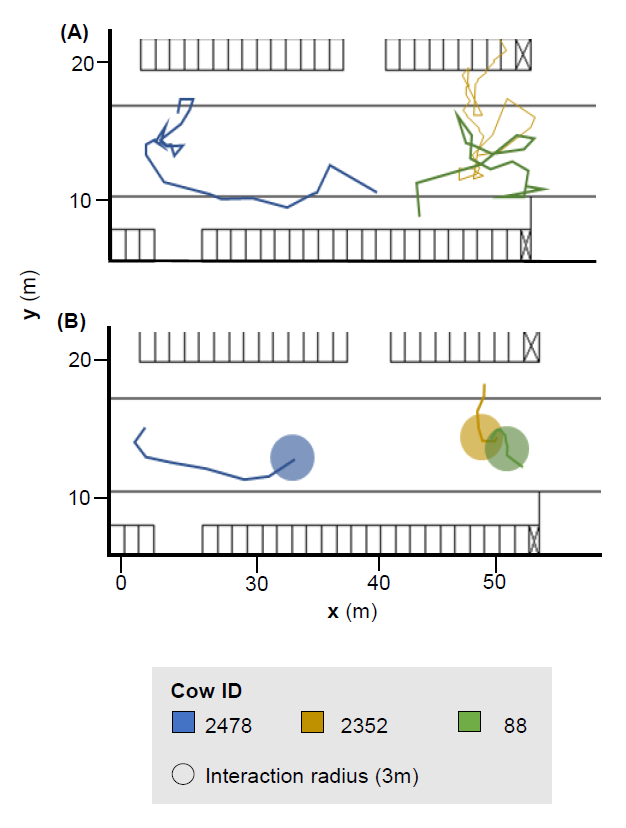


**Supplementary Material 2 Fig 3.** (A) Raw and (B) smoothed trajectory data (using a simple moving average with a window size of 15 data points) for three cows, coloured by identity: 4 = blue, 2352 = yellow and 88 = green, on 01/10/2014 between 15:18:30 to 15:23:30. The radius required to define an interaction (3m) is shown for each cow in (B). During this time period, Cow 2352 and 88 wereclassed as interacting, whereas cow 2478did not interact with any other cows.

*2.4 Further nonsensical data*

A semi-automated algorithm, created for the purposes of this study, was used to identify periods where the trajectory of a cow was located within a very small area (but not exactly the same coordinates as in Step 2 in Supplementary Material 2Fig 1) for an extended period of time (Step 4 in Supplementary Fig 1). Any such periods identified were then checked manually to determine if the apparent movement was biologically plausible, or if any farm management actions had taken place for that cow during the given time period; any implausible data was removed. The only instance found of this type was with sensor 000284 on day 05/10/2014. Visualising the trajectory of the cow deployed with this sensor (2585) on this dayimplies the cow only briefly entered the non-feeding zone, when in fact we know it must have spent most of the day there and was not subject to any farm management actions this day. Upon closer inspection, only 1101 data points were recorded for this cow in the non-feeding zone on this day, and a total of 2187 data points throughout the day, whereas the average points collected across all cows in the non-feeding zone alone was 6273 (*n* = 91). The data for cow 2585 on this day was therefore removed from the analysis (removing 0.01% of the original data; Supplementary Material 2Fig 4).


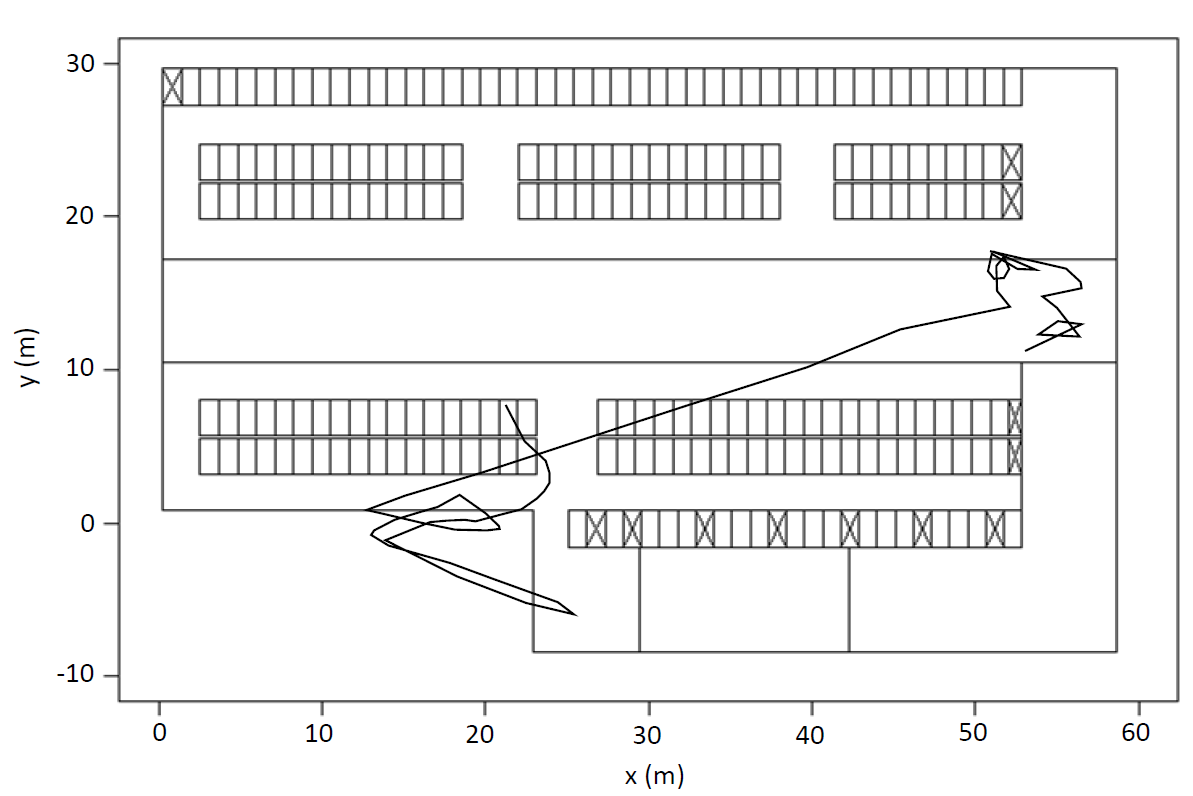


**Supplementary Material 2 Fig 4.**Trajectory plot showing data removed during step 4 of data cleaning. Sensor 000284 (cow 2585) on 05/10/2014 was only shown to move within a very small area throughout the entire day. There were no further instances of any sensors with this type of nonsensical location data within the study period.
