# Supplementary material for: Proximity Interactions in a Permanently Housed Dairy Herd: Network Structure, Consistency, and Individual Differences
Source: Front Vet Sci. 2020 Dec 7;7:583715. doi: 10.3389/fvets.2020.583715 (PMC7750390; doi:10.3389/fvets.2020.583715)
Supplement: Data Sheet 3 — Validation of proximity identification protocol and additional results for different time durations and spatial thresholds, and temporal segmentation. We test and validate our algorithm for identifying and classifying proximity interactions against observed proximity events across a range of parameters (spatial threshold radii, r = 1–5 m; time duration, t = 20-160 s). We include additional results similar to the main paper for these additional parameter values, as well as alternative formats of temporal segmentation of the 28-day study period. In all cases, the results are qualitatively similar to the results given in the main paper and our conclusions hold. [file Data_Sheet_3.docx]

***Supplementary Material 3***

# Validation of proximity identification algorithm and parameter choice

*1.1 Validation*

To validate our algorithm for identifying and classifying proximityinteractions, an observer (HH) recorded (*n* = 35) verified instances of cows lying in adjacent cubicles within a localized area of the barn (non-feeding zone) on five different dates before, during, and after the main study period 08/08/2014, 22/08/2014, 23/09/2014, 21/10/2014 and 18/11/2014. Each of these verified proximity interactions lastedon average ten minutes. A one-minute buffer was added around the start and end times of each observation (in case of any minor time-sync issues between observer and sensor clocks) before comparing them to proximity interactions detected from the sensor-collected location data using our automated algorithm.

As described in the main paper, the algorithm uses a ‘strict’ protocol to identify proximity interactions based on pairs of individual cows being within a specified radius (spatial threshold, *r*) for all time points within a sustained time period (duration, *t*). To test the sensitivity of the algorithm to the (arbitrary) choice of parameters, we considered classification performance over a range of spatial thresholds (*r* =1-5 m) andtime duration (*t* = 20-160s; 2-16 time points at 0.1Hz). Each of the verified observedproximity interactionswere either marked as a true positive (TP), where the algorithmreported a proximity interaction coinciding with an observation, or a false negative(FN) where the algorithm did not detect a proximity interaction when one had been observed. The sensitivity, or true positive rate (TPR), was then defined as TPR = TP / (TP + FN). Note that the nature of the observation process (identifying those cows lying in adjacent cubicles such that proximity interactions could be verified) meant that we did not record observations where cows were not interacting; hence we were not able to estimate the true negative (TN) or false positive (FP) rates, or the associated specificity (true negative rate, TNR).

Supplementary Material 3 Table 1 gives the sensitivity results for our proximity identification algorithm based on the ‘strict’ protocol described above. As might be expected, using a higher spatial threshold, *r*,anda lower time duration, *t*, increased the sensitivity, up to 0.97 (5m and 20-100s), while using a very small spatial threshold (1m) and a longer time duration (160s) reduced sensitivity to as low as 0.03.

The stringency of the algorithm can be reduced by identifying a proximity interaction when only a certain percentage of points within the time period, *t*, are within the specified spatial threshold, *r*. For example, classifying an interaction as when sensors are within a 3 m proximity for at least 50 % of the time points within an 80 s window (8 time points) increased the sensitivity to 0.89 (from 0.83 with the strict protocol). However, reducing the stringency of the algorithm would have almost certainly increased the false positive rate, while offering only minimal increases in sensitivity based on the validation results in Supplementary Material 3 Table 1.

**Supplementary Material 3 Table 1.**Sensitivity of proximity interaction algorithm based on verified observations across five days in August-November 2014 (*n* = 35, average duration often minutes). The sensitivity(TPR = TP/ (TP + FN)) is given for different combinations of spatial thresholds (*r* = 1-5 m) and time duration (*t* = 20-160 s).In all these cases, we used a ‘strict’ identification protocol: the inter-cow distance must be less than the spatial threshold, *r*, for all points throughout the time period, *t*, in order for a proximity interaction to be identified. The protocol based on a spatial threshold of *r* = 3m and time duration of *t* = 60s (indicated in bold) was chosen for use in the algorithm based on acceptable sensitivity (TPR = 0.83), expected lower rate of false positives, and to fit with practical considerations such as the size of an individual cow, the sensor location error, and the typical minimum duration of important social interactions.

| Time (s) | | | | | | | | |  |
| --- | --- | --- | --- | --- | --- | --- | --- | --- | --- |
|  | | 20 | 40 | **60** | 80 | 100 | 120 | 140 | 160 |
| Radius (m) | 1 | 0.46 | 0.31 | 0.29 | 0.17 | 0.14 | 0.09 | 0.06 | 0.03 |
|  | 2 | 0.71 | 0.66 | 0.63 | 0.63 | 0.49 | 0.46 | 0.37 | 0.23 |
|  | **3** | 0.86 | 0.86 | **0.83** | 0.83 | 0.74 | 0.71 | 0.69 | 0.53 |
|  | 4 | 0.91 | 0.91 | 0.89 | 0.86 | 0.86 | 0.80 | 0.77 | 0.74 |
|  | 5 | 0.97 | 0.97 | 0.97 | 0.97 | 0.97 | 0.91 | 0.86 | 0.86 |

*1.2 Parameter choice*

Ideally, we would have chosen parameters for the algorithm that maximized both the sensitivity and specificity. However, as we were not able to estimate specificity, we selected parameters based on a number of considerations. Basic logic dictates that using a larger spatial threshold or reducing the time duration would have increased false positives, where cows were a larger distance apart or only briefly passing each other are classified as interacting. Conversely, Supplementary Material 3 Table 1 illustrates how using a very small spatial threshold, or too long a time duration, significantly reduced the sensitivity of the algorithm. In addition, there are practical considerations to take into account: the location sensors have previously been reported to have a mean error distance of 2.66m in this barn environment (1,2); the typical body length of a dairy cow is 2.3-2.6m; we were also mainly interested in sustained proximity interactions that were not simply incidental ‘passing’ interactions. Hence, to balance the validated sensitivity, our expectations about how specificity would relate to parameter choice, and the practical considerations mentioned, we chose to adopt a protocol based on a spatial threshold of *r* = 3m and a time duration of *t* = 60s.

Our choice of time duration compares to other recent studies on dairy cow social interactions that use thresholds ranging from 2s (3)to 10 minutes (4), and is similar to (5) where a minimum of 6 time points within a window of 12 consecutive time points [0.1 Hz] was used (alongside a spatial threshold of two meters). Previous studies have also used similar spatial thresholds to detect cattle interactions, ranging from *r* = 1.5 to 4 m (3–6).

To further test the logic behind our choice of parameters, we ran the algorithm over a range of spatial thresholds and time durations with the full herd data (all dyad parings) for individual days within the study period. Supplementary Material 3 Fig 1A illustrates how using a short time duration dramatically increased the number of proximity interactions (92664interactions at 20s, compared to 58499interactions at 60s, or 37628interactions at 160s).Supplementary Material 3 Fig 1B illustrates how increasing the spatial threshold resulted in an increased number of proximity interactions (214,813interactions at 5m, compared to 76,330 interactions at 3m, and 5,393 interactions at 1m).


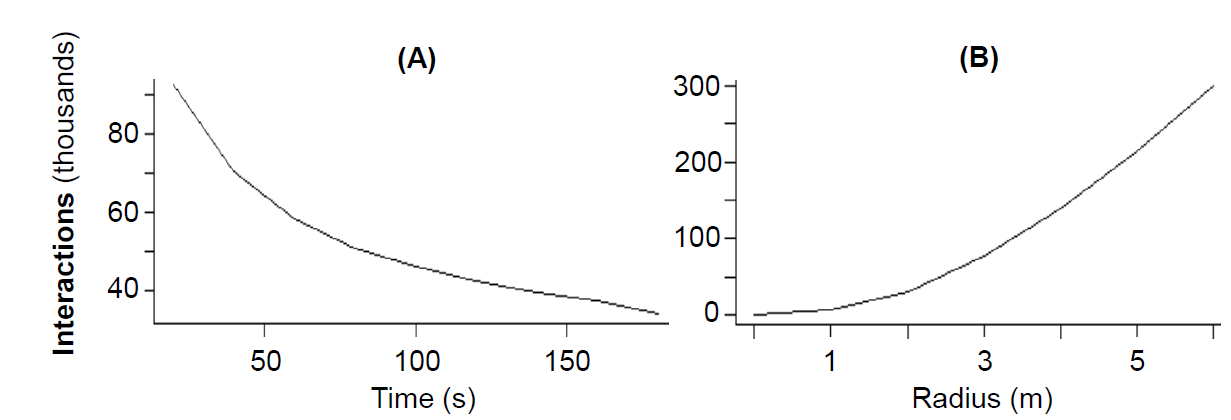


**Supplementary Material 3 Figure1.** Interactions identified between all (4186) dyads when running the algorithm on an example day, 01/10/2014 (results are very similar for other days) for (A) different time durations (*t* = 20s – 180s; 2-18 recorded at 0.1 Hz; increments of 20s or 2 time points) and for (B) different spatial threshold radii (*r* = 0-6 m, increments of one metre).

# Additional results for different spatial thresholds

To fully explore the effect of the spatial threshold radius,*r*, additional full network analyses were run using one, two, four and five meters for a fixed time duration of *t* = 60s. In all cases, qualitatively similar results were obtained, including significant social differentiation, high temporal variation in the network structure, and no social assortment by lameness, parity or DIM (seeSupplementary Material 3 Tables 2-5, respectively), demonstrating that our overall results and conclusions are robust to this parameter choice.

To fully explore the effect of the time duration threshold, *t*, additional full network analyses were run using 40, 80 and 100s for a fixed radius of *r* = 3 m. In all cases, qualitatively similar results wereobtained, including significant social differentiation, high temporal variation in the network structure, and no social assortment by lameness, parity or DIM (see Supplementary Material 3 Tables 6-8, respectively), demonstrating that our overall results and conclusions are robust to this parameter choice.

**Supplementary Material 3Table 2.** Overview ofresults using a spatial threshold radius of *r* = 1 m and time duration of *t* = 60s to define an interaction for the full barn (FB) and the functional zones: feeding zone (FZ) and non-feeding zone (NFZ): basic network measures (original and filtered by mean degree), inter-individual variation, temporal variation in sociality, lameness status, and parity and days in milk, where (M)DI = (median) daily interactions. Significant results (*p*< 0.05) are in bold.

|  | **Measure** | **Test value** (*p-*value) | | | **Summary** |
| --- | --- | --- | --- | --- | --- |
|  |  | **Full barn** | **Feeding zone** | **Non-feeding zone** |  |
| Basic network measures | Mean edge density  (*d* = 28) | 0.46 | 0.04 | 0.43 | The networks are highly dense, more so the NFZ than the FZ. |
|  | Components  (by day) (*d* = 28) | 1 | 6-19 | 1 | The full barn and the NFZ consisted of one component but the FZ consisted of numerous. |
| Inter-individual variation | Inter-individual differences in median DI (*n* = 92) | K-W = 36.06  (*p* = 1) | K-W = 701.83  (*p =*1) | K-W = 31.24  **(*p <*0.001)** | **Inter-individual variation in DI in the NFZ** but not in the full barn or the FZ. |
|  | Social differentiation (SD) (*n* = 92) | SD between >100 %  of dyads  **(*p* <0.01)** | SD between 100 %  of dyads  **(*p* <0.01)** | SD between100 %  of dyads  **(*p* < 0.01)** | **Social differentiation present in all networks.** |
| Temporal variation in sociality | Difference in medianDI between days(*n* = 92, d = 28) | K-W =2154.40  (*p* = 1) | K-W = 43.65  (*p* = 0.98) | K-W = 2162.90  (*p* = 1) | No difference in DI between days in all networks. |
|  | Relationship between MDI and days (*n* = 92, *d* = 28) | Pearson correlation,  *ρ* = -0.01  (*p* = 0.97) | Pearson correlation,  *ρ*= 0.28  *(p =*0.15) | Pearson correlation,  * *= -0.01  (*p* ***=***0.96) | MDI weakly correlated over time in both functional zones. |
|  | Relationship between MDI and temperature (*n* = 92, *d* = 28) | Pearson correlation,  *ρ* = -0.04  (*p* = 0.84) | Pearson correlation,  *ρ* = -0.23  (*p* = 0.24) | Pearson correlation,  *ρ* = -0.04  (*p* =0.85) | Weak negative correlation between MDI and temperature in both functional zones. |
|  | Relationship between four-day block consecutive networks (six networks, *n* = 92 per network) | Mantel test,  range of R_s_ = 0.05to 0.34  (***p<*0.01)**for five comparisons(day blocks 1-2, 2-3, 3-4, 4-5, 5-6);  R_s_ = -0.14  *(p* = 1) for one comparison (day block 6-7) | Mantel test,  range of R_s_ = 0.05 to 0.14  (***p ≤* 0.01)**for five comparisons (1-2, 2-3, 3-4; 4-5, 6-7);  R_s =_ *=* 0.02  (*p* = 0.54) for one comparison(day block 5-6) | Mantel test,  range of R_s_ = 0.05 to 0.35  (***p* < 0.01)** for five comparisons (day blocks 1-2, 2-3, 3-4, 4-5, 5-6);  R_s_= -0.15  (*p*= 1) for one comparison(day block 6-7) | Weak correlation between all consecutive networks. |
| Individual characteristics | Difference in meanDI between non-lame (*n* = 26) and lame cows (*n* = 22) | Wilcoxon test,  W = 255  (*p* =0.25) | Wilcoxon test,  W = 317  (*p* =0.72) | Wilcoxon test,  W = 237  (*p =* 0.15) | No difference in DI between non-lame and lame cows in both functional zones. |
|  | Difference in mean clustering coefficient between non-lame (*n* = 26) and lame cows (*n* = 22) | Wilcoxon test,  W = 419  (*p* =1) | Wilcoxon test,  W = 262.50  (*p*= 0.30**)** | Wilcoxon test,  W = 417  (*p* = 1) | No difference in clustering coefficient between non-lame and lame cows in either functional zone. |
|  | Difference in mean node degree between non-lame (*n* = 26) and lame cows (*n* = 22) | Wilcoxon test,  W = 261.50  (*p* =0.29) | Wilcoxon test,  W = 305.50  (*p* =0.64) | Wilcoxon test,  W = 210  (*p* = 0.05) | No difference in node degree between non-lame and lame cows in either functional zone. |
|  | Social assortment bylameness status by day, *n* = 48) | Mantel test,  range of R_s_= -0.07 to 0.07  (*p*> 0.34 for all days) | Mantel test,  range of R_s_ = -0.06 to0.06  (*p* > 0.70for all days) | Mantel test,  range of R_s_ = -0.07 to 0.07  (*p ≥* 0.35) | Cows did not socially assort according to their lameness status, parity, or DIM in either functional zone. |
|  | Social assortment by parity (by day, *n* = 92) | Mantel test,  range of R_s_ = -0.03 to 0.02  (*p* =1 for all days) | Mantel test,  range of R_s_ = -0.04 to 0.02  (*p* =1 for all days) | Mantel test,  range of R_s_= -0.02 to 0.03  (*p* =1 for all days) |  |
|  | Social assortmentby DIM (by day, n = 92) | Mantel test,  range of R_s_ = -0.02 to 0.02  (*p* =1 for all days) | Mantel test,  range of R_s_ = -0.04 to 0.03  (*p* =1 for all days) | Mantel test,  range of R_s_ = -0.02 to 0.03  (*p =* 1 for all days) |  |

**Supplementary Material 3Table 3.** Overview ofresults using a spatial threshold radius of *r* = 2 m and time duration of *t* = 60s to define an interaction for the full barn (FB) and the functional zones: feeding zone (FZ) and non-feeding zone (NFZ): basic network measures (original and filtered by mean degree), inter-individual variation, temporal variation in sociality, lameness status, and parity and days in milk, where (M)DI = (median) daily interactions. Significant results (*p*< 0.05) are in bold.

|  | **Measure** | **Test value** (*p-*value) | | | **Summary** |
| --- | --- | --- | --- | --- | --- |
|  |  | **Full barn** | **Feeding zone** | **Non-feeding zone** |  |
| Basic network measures | Mean edge density  (*d* = 28) | 0.84 | 0.19 | 0.80 | The networks are highly dense, more so the NFZ than the FZ. |
|  | Components  (by day) (*d* = 28) | 1 | 1-4 | 1 | The networks typically consist of one component. |
| Inter-individual variation | Inter-individual differences in median DI (*n* = 92) | K-W = 29.22  **(*p <*0.001)** | K-W = 928.39  (*p =* 1) | K-W = 21.57  **(*p* < 0.001)** | **Inter-individual variation in DI in the NFZ**but not in the FZ. |
|  | Social differentiation (SD) (*n* = 92) | SD between >96.43 %  of dyads  **(*p* <0.01)** | SD between 100 %  of dyads  **(*p* <0.01)** | SD between96.46 %  of dyads  **(*p* < 0.01)** | **Social differentiation present in all networks.** |
| Temporal variation in sociality | Difference in medianDI between days(*n* = 92, d = 28) | K-W =2282.10  (*p* = 1) | K-W = 49.47  (*p* = 0.99) | K-W = 2303.00  (*p* = 1) | No difference in DI between days in all networks. |
|  | Relationship between MDI and days (**n** = 92, *d* = 28) | Pearson correlation,  * *= -0.03  (*p* = 0.87) | Pearson correlation,  *ρ* = 0.32  *(p =* 0.09) | Pearson correlation,  *ρ* = -0.04  (*p* ***=***0.86) | MDI weakly correlated over time in both functional zones. |
|  | Relationship between MDI and temperature (*n* = 92, *d* = 28) | Pearson correlation,  * *= -0.04  (*p* = 0.83) | Pearson correlation,  *ρ* = -0.19  (*p* = 0.34) | Pearson correlation,  *ρ* = -0.04  (*p* =0.83) | Weak negative correlation between MDI and temperature in both functional zones. |
|  | Relationship between four-day block consecutive networks (six networks, *n* = 92 per network) | Mantel test,  range of R_s_ = 0.04to 0.26  **(*p ≤* 0.01)**for five comparisons(day blocks 2-3, 3-4, 4-5, 5-6, 6-7);  R_s_ = -0.10 (*p* = 1) for one comparison (day block 1-2) | Mantel test,  range of R_s_ = 0.10to 0.25  **(*p <*0.001**) | Mantel test,  range of R_s_ = 0.05 to 0.28  (*p ≤* 0.10)for five comparisons (day blocks 2-3, 3-4, 4-5, 5-6, 6-7);  R_s_ = -0.10 (*p*= 1) for one comparison (day block 1-2) | Weak correlation between all consecutive networks. |
| Individual characteristics | Difference in meanDI between non-lame (*n* = 26) and lame cows (*n* = 22) | Wilcoxon test,  W = 272.00  (*p* =0.37) | Wilcoxon test,  W = 311.50  (*p* = 0.68) | Wilcoxon test,  W = 236.50  (*p =* 0.14) | No difference in DI between non-lame and lame cows in both functional zones. |
|  | Difference in mean clustering coefficient between non-lame (*n* = 26) and lame cows (*n* = 22) | Wilcoxon test,  W = 397.00  (*p* =0.99) | Wilcoxon test,  W = 290.00  (*p* = 0.51) | Wilcoxon test,  W = 412.00  (*p* = 0.99) | No difference in clustering coefficient between non-lame and lame cows in either functional zone. |
|  | Difference in mean node degree between non-lame (*n* = 26) and lame cows (*n* = 22) | Wilcoxon test,  W = 306.50  (*p* =0.65) | Wilcoxon test,  W = 299  (*p* = 0.59) | Wilcoxon test,  W = 250.50  (*p* = 0.22) | No difference in node degree between non-lame and lame cows in either functional zone. |
|  | Social assortment bylameness status by day, *n* = 48) | Mantel test,  R_s_= -0.06 to 0.09  (*p*≥ 0.06)for all days | Mantel test,  range of R_s_ = -0.04 to 0.06  (*p* ≥ 0.46for all days) | Mantel test,  range of R_s_ = -0.07 to 0.07  (*p* > 0.38) | Cows did not socially assort according to their lameness status, parity, or DIM in either functional zone. |
|  | Social assortment by parity (by day, *n* = 92) | Mantel test,  range of R_s_ = -0.03 to 0.02  (*p* =1 for all days) | Mantel test,  range of R_s_ = -0.05 to 0.04  (*p* =1 for all days) | Mantel test,  range of R_s_ = -0.02 to 0.03  (*p* =1 for all days) |  |
|  | Social assortmentby DIM (by day, *n* = 92) | Mantel test,  range of R_s_ = -0.03 to 0.02  (*p* =1 all days) | Mantel test,  range of R_s_ = -0.04 to 0.02  (*p* =1 for all days) | Mantel test,  range of R_s_ = -0.03 to 0.03  (*p* ≥ 0.87 for all days) |  |

**Supplementary Material 3Table 4.** Overview ofresults using a spatial threshold radius of *r* = 4 m and time duration of *t* = 60s to define an interaction for the full barn (FB) and the functional zones: feeding zone (FZ) and non-feeding zone (NFZ): basic network measures (original and filtered by mean degree), inter-individual variation, temporal variation in sociality, lameness status, and parity and days in milk, where (M)DI = (median) daily interactions.. Significant results (*p*< 0.05) are in bold.

|  | **Measure** | **Test value** (*p-*value) | | | **Summary** |
| --- | --- | --- | --- | --- | --- |
|  |  | **Full barn** | **Feeding zone** | **Non-feeding zone** |  |
| Basic network measures | Mean edge density (*d* = 28) | 0.99 | 0.49 | 0.99 | The networks are highly dense, more so the NFZ than the FZ. |
|  | Components  (by day) (*d* = 28) | 1 | 1-3 | 1 | The networks typically consist of one component. |
| Inter-individual variation | Inter-individual differences in median DI (*n* = 92) | K-W = 18.49  (*p =*1) | K-W = 1001.70  (*p =* 1) | K-W = 9.76  **(*p <*0.001)** | **Inter-individual variation in DI in the NFZ**but not in the NFZ. |
|  | Social differentiation (SD) (*n* = 92) | SD between ≥ 78.76 %  of dyads  **(*p* <0.01)** | SD between ≥ 99.95 % of dyads  **(*p* <0.01)** | SD between ≥78.76 %  of dyads  **(*p* < 0.01)** | **Social differentiation present in all networks.** |
| Temporal variation in sociality | Difference in medianDI between days(*n* = 92, *d* = 28) | K-W =2353.70  (*p* = 1) | K-W = 63.41  (*p* = 1) | K-W = 2376.40  (*p* = 1) | No difference in DI between days in all networks. |
|  | Relationship between MDI and days (*n* = 92, *d* = 28) | Pearson correlation,  *ρ* = -0.05  (*p* = 0.81) | Pearson correlation,  *ρ* = 0.34  *(p =* 0.07) | Pearson correlation,  *ρ* = -0.11  (*p*=0.28) | MDI weakly correlated over time in both functional zones. |
|  | Relationship between MDI and temperature (*n* = 92, *d* = 28) | Pearson correlation,  *ρ* = -0.11  (*p* = 0.59) | Pearson correlation,  *ρ* = -0.10  (*p* = 0.62) | Pearson correlation,  *ρ* = -0.55  (*p* =0.79) | Weak negative correlation between MDI and temperature in both functional zones. |
|  | Relationship between four-day block consecutive networks (six networks, *n* = 92 per network) | Mantel test,  range of R_s_ = 0.09to 0.25  **(*p* < 0.001)**for four comparisons (day blocks 3-4, 4-5, 5-6, 6-7);  range of R_s_ = -0.26 to 0.03  (*p*≥ 0.09) for two comparisons (day blocks 1-2, 2-3) | Mantel test,  range of R_s_ = 0.21 to 0.44  **(*p <*0.001**) | Mantel test,  range of R_s_ = 0.07 to 0.25  **(*p*< 0.001)**for four comparisons (day blocks 3-4, 4-5, 5-6, 6-7);  range of R_s_ = -0.34 to -0.003  (*p*= 1) for two comparisons  (day blocks 1-2, 2-3) | Weak correlationbetween all consecutive networks. |
| Individual characteristics | Difference in meanDI between non-lame (*n* = 26) and lame cows (*n* = 22) | Wilcoxon test,  W = 286.00  (*p* =0.49) | Wilcoxon test,  W = 315.50  (*p* =0.71) | Wilcoxon test,  W = 271.00  (*p =* 0.35) | No difference in DI between non-lame and lame cows in both functional zones. |
|  | Difference in mean clustering coefficient between non-lame (*n* = 26) and lame cows (*n* = 22) | Wilcoxon test,  W = 325.00  (*p* =0.79) | Wilcoxon test,  W = 301.00  (*p*= 0.61) | Wilcoxon test,  W = 374.00  (*p* = 0.96) | No difference in clustering coefficient between non-lame and lame cows in either functional zone. |
|  | Difference in mean node degree between non-lame (*n* = 26) and lame cows (*n* = 22) | Wilcoxon test,  W = 321.50  (*p* =0.75) | Wilcoxon test,  W = 295.50  (*p* =0.56) | Wilcoxon test,  W = 245.50  (*p* = 0.18) | No difference in node degree between non-lame and lame cows in either functional zone. |
|  | Social assortment bylameness status by day, *n* = 48) | Mantel test,  R_s_ = 0.10  (***p* = 0.01)**for day 26;  range of R_s_= -0.06 to 0.06  (p ≥ 0.89) for 27 days | Mantel test,  range of R_s_ = -0.07 to0.06  (*p ≥* 0.37 for all days) | Mantel test,  range of R_s_ = -0.07 to 0.07  (*p* ≥ 0.39) | Cows did not socially assort according to their lameness status, parity, or DIM in either functional zone. |
|  | Social assortment by parity (by day, *n* = 92) | Mantel test,  range of R_s_ = -0.02 to 0.03  (*p* =1 for all days) | Mantel test,  range of R_s_ = -0.04 to 0.03  (*p* =1 for all days) | Mantel test,  range of R_s_ = -0.02 to 0.03  (*p ≥* 0.84 for all days) |  |
|  | Social assortmentby DIM (by day, *n* = 92) | Mantel test,  range of R_s_ = -0.03 to 0.03  (*p* =1 for all days) | Mantel test,  range of R_s_ = -0.04 to 0.03  (*p* =1 for all days) | Mantel test,  range of R_s_ = 0.02 to 0.04  (*p ≥* 0.45 for all days) |  |

**Supplementary Material 3Table 5.** Overview ofresults using a spatial threshold radius of *r* = 5 m and time duration of *t* = 60s to define an interaction for the full barn (FB) and the functional zones: feeding zone (FZ) and non-feeding zone (NFZ): basic network measures (original and filtered by mean degree), inter-individual variation, temporal variation in sociality, lameness status, and parity and days in milk, where (M)DI = (median) daily interactions. Significant results (*p*< 0.05) are in bold.

|  | **Measure** | **Test value** (*p-*value) | | | **Summary** |
| --- | --- | --- | --- | --- | --- |
|  |  | **Full barn** | **Feeding zone** | **Non-feeding zone** |  |
| Basic network measures | Mean edge density (*d* = 28) | 1 | 0.59 | 1.00 | The networks are highly dense, more so the NFZ than the FZ. |
|  | Components  (by day) (*d* = 28) | 1 | 1-3 | 1 | The networks typically consist of one component. |
| Inter-individual variation | Inter-individual differences in median DI (*n* = 92) | K-W = 21.78  (*p =*1) | K-W = 1018.60  (*p =* 1) | K-W = 11.98  **(*p <*0.001)** | **Inter-individual variation in DI in the NFZ**but not in the NFZ. |
|  | Social differentiation (SD) (*n* = 92) | SD between ≥ 70.91 %  of dyads  **(*p* <0.01)** | SD between ≥ 99.95 %  of dyads  **(*p* <0.01)** | SD between ≥ 70.60 %  of dyads  **(*p <* 0.01)** | **Social differentiation present in all networks.** |
| Temporal variation in sociality | Difference in medianDI between days(*n* = 92, *d* = 28) | K-W =2370.70  (*p* = 1) | K-W = 65.58  (*p* = 1) | K-W = 2394.40  (*p* = 1) | No difference in DI between days in all networks. |
|  | Relationship between MDI and days (*n* = 92, *d* = 28) | Pearson correlation,  * *= -0.06  (*p* = 0.76) | Pearson correlation,  *ρ* = 0.33  *(p =* 0.08) | Pearson correlation,  * *= -0.06  (*p* ***=***0.74) | MDI weakly correlated over time in both functional zones. |
|  | Relationship between MDI and temperature (*n* = 92, *d* = 28) | Pearson correlation,  *ρ* = -0.15  (*p* = 0.44) | Pearson correlation,  *ρ* = -0.07  (*p* = 0.73) | Pearson correlation,  *ρ* = -0.15  (*p* = 0.44) | Weak negative correlation between MDI and temperature in both functional zones. |
|  | Relationship between four-day block consecutive networks (six networks, *n* = 92 per network) | Mantel test,  range of R_s_ = 0.04 to 0.24  **(*p ≤* 0.02)**for five comparisons (day blocks 2-3, 3-4, 4-5, 5-6, 6-7);  R_s_ = -0.26  (*p* = 1) for one comparison (day block 1-2) | Mantel test,  range of R_s_ = 0.23 to 0.50  **(*p <*0.001**) | Mantel test,  range of R_s_ = 0.19 to 0.25  **(*p*** *<***0.001)**for four comparisons; (day blocks 2-3, 4-5, 5-6, 5-7);  range of R_s_ = -0.33 to 0.01  (*p* = 1) for two comparisons (day blocks 1-2, 3-4) | Weak correlation between all consecutive networks. |
| Individual characteristics | Difference in meanDI between non-lame (*n* = 26) and lame cows (*n* = 22) | Wilcoxon test,  W = 292.00  (*p* =0.52) | Wilcoxon test,  W = 312.50  (*p* =0.69) | Wilcoxon test,  W = 277  (*p =* 0.40) | No difference in DI between non-lame and lame cows in both functional zones. |
|  | Difference in mean clustering coefficient between non-lame (*n* = 26) and lame cows (*n* = 22) | Wilcoxon test,  W = 266.00  (*p* = 0.33) | Wilcoxon test,  W = 297  (*p****=*** 0.56) | Wilcoxon test,  W = 343.00  (*p* = 0.87) | No difference in clustering coefficient between non-lame and lame cows in either functional zone. |
|  | Difference in mean node degree between non-lame (*n* = 26) and lame cows (*n* = 22) | Wilcoxon test,  W = 337.50  (*p* =0.85) | Wilcoxon test,  W = 303.50  (*p* =0.62) | Wilcoxon test,  W = 267.50  (*p* = 0.34) | No difference in node degree between non-lame and lame cows in either functional zone. |
|  | Social assortment bylameness status by day, *n* = 48) | Mantel test,  R_s_ = 0.10  **(*p*= 0.02)**for day 26;  range of R_s_= -0.06 to 0.06  *(p* ≥ 0.58) for 27 days | Mantel test,  range of R_s_ = -0.06 to 0.06  (*p ≥* 0.48for all days) | Mantel test,  range of R_s_ = -0.06 to 0.08  (*p ≥* 0.17) | Cows did not socially assort according to their lameness status, parity, or DIM in either functional zone. |
|  | Social assortment by parity (by day, *n* = 92) | Mantel test,  range of R_s_ = -0.02 to 0.03  (*p* =1 for all days) | Mantel test,  range of R_s_ = -0.05 to 0.04  (*p* =1 for all days) | Mantel test,  range of R_s_ = -0.01 to 0.03  (*p* =1 for all days) |  |
|  | Social assortmentby DIM (by day, *n* = 92) | Mantel test,  range of R_s_ = -0.03 to 0.03  (*p* =1 for all days) | Mantel test,  range of R_s_ = -0.04 to 0.03  (*p* =1for all days) | Mantel test,  range of R_s_= -0.03 to 0.03  (*p* ≥ 0.59 for all days) |  |

**Supplementary Material 3Table 6.** Overview ofresults using a spatial threshold radius of *r* = 3 m and time duration of *t* = 40s to define an interaction for the full barn (FB) and the functional zones: feeding zone (FZ) and non-feeding zone (NFZ): basic network measures (original and filtered by mean degree), inter-individual variation, temporal variation in sociality, lameness status, and parity and days in milk, where (M)DI = (median) daily interactions. Significant results (*p*< 0.05) are in bold.

|  | **Measure** | **Test value** (*p-*value) | | | **Summary** |
| --- | --- | --- | --- | --- | --- |
|  |  | **Full barn** | **Feeding zone** | **Non-feeding zone** |  |
| Basic network measures | Mean edge density (*d* = 28) | 0.99 | 0.52 | 0.98 | The networks are highly dense, more so the NFZ than the FZ. |
|  | Components  (by day) (*d* = 28) | 1 | 1-3 | 1 | The networks typically consist of one component. |
| Inter-individual variation | Inter-individual differences in median DI (*n* = 92) | K-W = 20.90  **(*p <*0.001)** | K-W = 1001.30  (*p* = 1) | K-W = 12.94  **(*p <* 0.001)** | **Inter-individual variation in DI in the NFZ**but not in the NFZ. |
|  | Social differentiation (SD) (*n* = 92) | SD between ≥ 82.30 %  of dyads  **(*p* <0.01)** | SD between 100 %  of dyads  **(*p* <0.01)** | SD between ≥ 83.30 %  of dyads  **(*p* < 0.01)** | **Social differentiation present in all networks.** |
| Temporal variation in sociality | Difference in medianDI between days(*n* = 92, *d* = 28) | K-W =2340.10  (*p* = 1) | K-W = 57.27  (*p* = 1) | K-W = 2358.10  (*p* = 1) | No difference in DI between days in all networks. |
|  | Relationship between MDI and days (*n* = 92, *d* = 28) | Pearson correlation,  *ρ* = -0.06  (*p* = 0.74) | Pearson correlation,  *ρ* = 0.27  *(p =* 0.16) | Pearson correlation,  *ρ* = -0.07  (*p* ***=***0.73) | MDI weakly correlated over time in both functional zones. |
|  | Relationship between MDI and temperature (*n* = 92, *d* = 28) | Pearson correlation,  * *= -0.06  (*p* = 0.76) | Pearson correlation,  *ρ* = -0.13  (*p* = 0.52) | Pearson correlation,  *ρ* = -0.06  (*p* = 0.77) | Weak negative correlation between MDI and temperature in both functional zones. |
|  | Relationship between four-day block consecutive networks (six networks, *n* = 92 per network) | Mantel test,  range of R_s_ = 0.05 to 0.29  **(*p* < 0.01)**for five comparisons (day blocks 2-3, 3-4, 4-5, 5-6, 6-7);  R_s_= -0.25 (*p* = 1 for one comparison (day block 1-2) | Mantel test,  range of R_s_= 0.18 to 0.41  **(*p<*0.001**) | Mantel test,  range of R_s_ = -0.28 to 0.29  **(*p <*0.001)**for five comparisons (day blocks 2-3, 3-4, 4-5, 5-6, 6-7);  R_s_= -0.28  (*p* = 1) for one comparison (day block 1-2) | Weak correlation between all consecutive networks. |
| Individual characteristics | Difference in meanDI between non-lame (*n* = 26) and lame cows (*n* = 22) | Wilcoxon test,  W = 280.00  (*p* =0.42) | Wilcoxon test,  W = 305.50  (*p* =0.63) | Wilcoxon test,  W = 236.00  (*p* = 0.14) | No difference in DI between non-lame and lame cows in both functional zones. |
|  | Difference in mean clustering coefficient between non-lame (*n* = 26) and lame cows (*n* = 22) | Wilcoxon test,  W = 338.00  (*p* =0.85) | Wilcoxon test,  W = 270.00  (*p*= 0.35) | Wilcoxon test,  W = 375.00  (*p* = 0.96) | No difference in clustering coefficient between non-lame and lame cows in either functional zone. |
|  | Difference in mean node degree between non-lame (*n* = 26) and lame cows (*n* = 22) | Wilcoxon test,  W = 331.50  (*p* =0.81) | Wilcoxon test,  W = 293.00  (*p* =0.54) | Wilcoxon test,  W = 251.00  (*p* = 0.22) | No difference in node degree between non-lame and lame cows in either functional zone. |
|  | Social assortment bylameness status by day, *n* = 48) | Mantel test,  R_s_ = 0.09  **(*p* = 0.03)** for day 26;  range of R_s_ =-0.07 to 0.06  (*p* ≥ 0.84) for 27 days | Mantel test,  range of R_s_ = -0.06 to 0.05  (*p* ≥ 0.76for all days) | Mantel test,  range of R_s_ = -0.07 to 0.07  (*p ≥* 0.24) | Cows did not socially assort according to their lameness status, parity, or DIM in either functional zone. |
|  | Social assortment by parity (by day, *n* = 92) | Mantel test,  range of R_s_ = -0.02 to 0.02  (*p* =1for all days) | Mantel test,  range of R_s_= -0.05 to 0.02  (*p* =1 for all days) | Mantel test,  range of R_s_ = -0.02 to 0.03  (*p ≥* 0.97 for all days) |  |
|  | Social assortmentby DIM (by day, *n* = 92) | Mantel test,  range of R_s_ = -0.02 to 0.03  (*p*≥ 0.74 for all days) | Mantel test,  range of R_s_ = -0.05 to 0.02  (*p* = 1 for all days) | Mantel test,  range of R_s_ = -0.02 to 0.03  (*p ≥* 0.42 for all days) |  |

**Supplementary Material 3Table 7.** Overview ofresults using a spatial threshold radius of *r* = 3 m and time duration of *t* = 80s to define an interaction for the full barn (FB) and the functional zones: feeding zone (FZ) and non-feeding zone (NFZ): basic network measures (original and filtered by mean degree), inter-individual variation, temporal variation in sociality, lameness status, and parity and days in milk, where (M)DI = (median) daily interactions. Significant results (*p*< 0.05) are in bold.

|  | **Measure** | **Test value** (*p-*value) | | | **Summary** |
| --- | --- | --- | --- | --- | --- |
|  |  | **Full barn** | **Feeding zone** | **Non-feeding zone** |  |
| Basic network measures | Mean edge density (*d* = 28) | 0.93 | 0.23 | 0.91 | The networks are highly dense, more so the NFZ than the FZ. |
|  | Components  (by day) (*d* = 28) | 1 | 1-4 | 1 | The networks typically consist of one component. |
| Inter-individual variation | Inter-individual differences in median DI (*n* = 92) | K-W = 18.92  **(*p <* 0.001)** | K-W = 953.09  (*p =*1) | K-W = 13.94  **(*p <*0.001)** | **Inter-individual variation in DI in the NFZ** but not in the FZ. |
|  | Social differentiation (SD) (*n* = 92) | SD between > 100 %  of dyads  **(*p* < 0.01)** | SD between 99.98 %  of dyads  **(*p* < 0.01)** | SD between 82.30 %  of dyads  **(*p* < 0.01)** | **Social differentiation present in all networks.** |
| Temporal variation in sociality | Difference in medianDI between days(*n* = 92, *d* = 28) | K-W = 2327.80  (*p* = 1) | K-W = 57.29  (*p* = 1) | K-W = 2338.4  (*p =* 1) | No difference in DI between days in all networks. |
|  | Relationship between MDI and days (*n* = 92, *d* = 28) | Pearson correlation,  *ρ* = -0.05  (*p* = 0.79) | Pearson correlation,  *ρ* = 0.28  (*p* = 0.15) | Pearson correlation,  *ρ* = -0.06  (*p* = 0.80) | MDI not correlated over time in either functional zone. |
|  | Relationship between MDI and temperature (*n* = 92, *d* = 28) | Pearson correlation,  *ρ* = -0.04  (*p* = 0.86) | Pearson correlation,  *ρ* = -0.17  (*p =* 0.39) | Pearson correlation,  * *= -0.04  (*p =* 0.86) | Weak correlation between MDI  and temperature in both functional zones. |
|  | Relationship between four-day block consecutive networks (six networks, *n* = 92 per network) | Mantel test,  range of R_s_ = 0.03 to 0.31  **(*p ≤* 0.001)**for four comparisons (day blocks 3-4, 4-5, 5-6, 6-7);  range of R_s_ = -0.25 to 0.03  (*p* ≥ 0.18) for two comparisons (day blocks 1-2, 2-3) | Mantel test,  range of R_s_ = 0.12 to 0.30  **(*p* < 0.001)** | Mantel test,  range of R_s_ = -0.08 to 0.31  **(*p <*0.001)** for four comparisons (day blocks 3-4, 4-5, 5-6, 6-7); range of R_s_ = -0.28 to 0.02  (p ≥ 0.58) for two comparisons (day blocks 1-2, 2-3) | Weak correlation between all consecutive networks. |
| Individual characteristics | Difference in meanDI between non-lame (*n* = 26) and lame cows (*n* = 22) | Wilcoxon test,  W = 274.50  (*p* =0.39) | Wilcoxon test,  W = 317  (*p* =0.73) | Wilcoxon test,  W = 235.00  (*p* =0.14) | No difference in DI between non-lame and lame cows in both functional zones. |
|  | Difference in mean clustering coefficient between non-lame (*n* = 26) and lame cows (*n* = 22) | Wilcoxon test,  W = 429.00  (*p* =1) | Wilcoxon test,  W = 284.00  (*p* = 0.46) | Wilcoxon test,  W = 441  (*p* = 1) | No difference in clustering coefficient between non-lame and lame cows in either functional zone. |
|  | Difference in mean node degree between non-lame (*n* = 26) and lame cows (*n* = 22) | Wilcoxon test,  W = 308.00  (*p* =0.66) | Wilcoxon test,  W = 267.00  (*p* =0.33) | Wilcoxon test,  W = 274.50  (*p* = 0.39) | No difference in node degree between non-lame and lame cows in either functional zone. |
|  | Social assortment bylameness status by day, *n* = 48) | Mantel test,  range of R_s_= -0.07 to 0.08  (*p ≥* 0.14) | Mantel tests,  range of R_s_ = -0.04 to 0.08  (*p ≥* 0.07 for all days) | Mantel test,  range of R_s_= -0.07 to 0.07  (*p* ≥ 0.32) | Cows did not socially assort according to their lameness status, parity, or DIM in either functional zone. |
|  | Social assortment by parity (by day, *n* = 92) | Mantel test,  range of R_s_ = -0.02 to 0.03  (*p* = 1 for all days) | Mantel test,  range of R_s_ = -0.04 to 0.04  (*p* = 1 for all days) | Mantel test,  range of R_s_ = -0.02 to 0.03  (*p* =1 for all days) |  |
|  | Social assortmentby DIM (by day, *n* = 92) | Mantel test,  range of R_s_ = -0.02 to 0.03  (*p* =0.78for all days) | Mantel test,  range of R_s_ = -0.04 to 0.03  (*p* =1for all days) | Mantel test,  range of R_s_ = -0.02 to 0.03  (*p ≥* 0.41 for all days) |  |

**Supplementary Material 3Table 8.** Overview ofresults using a spatial threshold radius of *r* = 3 m and time duration of *t* = 100s to define an interaction for the full barn (FB) and the functional zones: feeding zone (FZ) and non-feeding zone (NFZ): basic network measures (original and filtered by mean degree), inter-individual variation, temporal variation in sociality, lameness status, and parity and days in milk, where (M)DI = (median) daily interactions. Significant results (*p* < 0.05) are in bold.

|  | **Measure** | **Test value** (*p-*value) | | | **Summary** |
| --- | --- | --- | --- | --- | --- |
|  |  | **Full barn** | **Feeding zone** | **Non-feeding zone** |  |
| Basic network measures | Mean edge density (*d* = 28) | 0.89 | 0.15 | 0.87 | The networks are highly dense, more so the NFZ than the FZ. |
|  | Components  (by day) (*d* = 28) | 1 | 1-5 | 1 | The networks typically consist of one component. |
| Inter-individual variation | Inter-individual differences in median DI (*n* = 92) | K-W = 16.79  **(*p <* 0.001)** | K-W = 937.39  (*p =* 1) | K-W = 13.95  **(*p <*0.001)** | **Inter-individual variation in DI in the NFZ**but not in the NFZ. |
|  | Social differentiation (SD) (*n* = 92) | SD between ≥ 85.63 %  of dyads  **(*p* <0.01)** | SD between 100 %  of dyads  **(*p* <0.01)** | SD between 99.98 %  of dyads  **(*p* < 0.01)** | **Social differentiation present in all networks.** |
| Temporal variation in sociality | Difference in medianDI between days(*n* = 92, *d* = 28) | K-W =2327.10  (*p* = 1) | K-W = 50.69  (*p* = 1) | K-W = 2332.50  (*p* = 1) | No difference in DI between days in all networks. |
|  | Relationship between MDI and days (*n* = 92, *d* = 28) | Pearson correlation,  *ρ* = -0.05  (*p* = 0.79) | Pearson correlation,  *ρ* = 0.28  *(p =* 0.14) | Pearson correlation,  *ρ* = -0.05  (*p* ***=***0.79) | MDI weakly correlated over time in both functional zones. |
|  | Relationship between MDI and temperature (*n* = 92, *d* = 28) | Pearson correlation,  * *= -0.03  (*p* = 0.88) | Pearson correlation,  *ρ* = -0.16  (*p* = 0.42) | Pearson correlation,  *ρ* = -0.03  (*p* = 0.88) | Weak negative correlation between MDI and temperature in both functional zones. |
|  | Relationship between four-day block consecutive networks (six networks, *n* = 92 per network) | Mantel test,  range of R_s_ = 0.06 to 0.31  ***(p ≤* 0.001)**for five comparisons (day block 2-3, 3-4, 4-5, 5-6, 6-7);  R_s_ *=* -0.24(*p* = 1) for one comparison (day block 1-2) | Mantel test,  range of R_s_ = 0.12 to 0.28  (***p ≤* 0.001** for all days) | Mantel test,  range of R_s_= 0.07 to 0.30  **(*p* < 0.001)**for five comparisons (day blocks 2-3, 3-4, 4-5, 5-6, 6-7);  R_s_ = -0.25  (*p* = 1) for one comparison (day block 1-2) | Weak correlation between all consecutive networks. |
| Individual characteristics | Difference in meanDI between non-lame (*n* = 26) and lame cows (*n* = 22) | Wilcoxon test,  W = 263.50  (*p* =0.30) | Wilcoxon test,  W = 315.00  (*p* =0.71) | Wilcoxon test,  W = 236.00  (*p =*0.14) | No difference in DI between non-lame and lame cows in both functional zones. |
|  | Difference in mean clustering coefficient between non-lame (*n* = 26) and lame cows (*n* = 22) | Wilcoxon test,  W = 420.00  (*p* =1) | Wilcoxon test,  W = 236.00  (*p =*0.15) | Wilcoxon test,  W = 436.00  (*p* = 1) | No difference in clustering coefficient between non-lame and lame cows in either functional zone. |
|  | Difference in mean node degree between non-lame (*n* = 26) and lame cows (*n* = 22) | Wilcoxon test,  W = 317.50  (*p* =0.73) | Wilcoxon test,  W = 315.00  (*p* =0.71) | Wilcoxon test,  W = 309.00  (*p* = 0.66) | No difference in node degree between non-lame and lame cows in either functional zone. |
|  | Social assortment bylameness status by day, *n* = 48) | Mantel test,  range of R_s_ = -0.06 to 0.08  (*p*≥ 0.17 for all days) | Mantel test,  range of R_s_ = -0.04 to 0.06  (*p* ***≥*** 0.60 for all days) | Mantel test,  range of R_s_= -0.07 to 0.07  (*p* ≥ 0.32) | Cows did not socially assort according to their lameness status, parity, or DIM in either functional zone. |
|  | Social assortment by parity (by day, *n* = 92) | Mantel test,  range of R_s_ = -0.02 to 0.03  (*p* = 1for all days) | Mantel test,  range of R_s_ = -0.03 to 0.05  (*p ≥* 0.96 for all days) | Mantel test,  range of R_s_ = -0.02 to 0.03  (*p* =1 for all days) |  |
|  | Social assortmentby DIM (by day, *n* = 92) | Mantel test,  range of R_s_ = -0.02 to 0.03  (*p* = 1for all days) | Mantel test,  range of R_s_ = -0.03 to 0.03  (*p* =1 for all days) | Mantel test,  range of R_s_ = -0.02 to 0.04  (*p ≥* 0.35 for all days) |  |

# Additional results for different temporal segmentation of study period

In the main paper, we assessed the temporal stability of the proximity interaction networks (feeding and non-feeding zone) by segmenting the 28-day study period into seven averaged four-day consecutive blocks, showing weak correlations, between networks in both functional zones (see Supplementary Material 3 Table 9). For completeness, we also compared each of the original daily networks (n = 28), as well as two-, seven-, and 14-day blocks. The results were qualitatively similar, showing weak correlations between all consecutive networks (Supplementary Material 3 Table 9).

**Supplementary Material 3 Table 9.** Temporal variation of the interaction network (interaction defined when cows (*n* = 92) were within a three meter radius for more than 60 seconds), by segmenting and averaging the networks (feeding and non-feeding zone) into one, two, seven and 14 day-blocks.To calculate the *p-*value, 10,000 permutations were used, and *p*-values are after Bonferroni correction; significant results (*p*< 0.05) are in bold.

| **Test value** (*p-*value) | |  |  |
| --- | --- | --- | --- |
| **Segmented components (days)** | **Feeding zone** | **Non-feeding zone** | **Summary** |
| 28 (1 day) | Range of R_s_ = 0.09 to 0.18  **(*p ≤* 0.01)**for 25 comparisons;  range of R_s_ = 0.02 to 0.04  (*p* =1) for two comparisons (days 21-22, 26-27) | Range of R_s_= -0.30 to 0.37  **(*p* ≤ 0.05)**for seven comparisons (days 3-4, 7-8, 13-14, 17-18, 18-19, 25-26, 27-28);  range of R_s_ = -0.28 to 0.03  (*p ≥* 0.42)for20 comparisons | Weak correlation between all consecutive networks. |
| 14 (2 days) | Range of R_s_ = 0.10to 0.26  (***p ≤* 0.01**) | Range of R_s_ = 0.06 to 0.29  (***p <*0.01**for six comparisons  (day blocks 2-3, 6-7, 10-11, 11-12, 12-13, 13-14);  range of R_s_ = -0.22 to 0.19  (*p* = 1) for seven comparisons |  |
| 7 (4 days) | Range of R_s_= 0.20 to 0.31  **(*p <* 0.001)** | Mantel test,  range of R_s_ = 0.05 to 0.24  **(*p <*0.01**) for four comparisons (day blocks 1-2, 2-3, 5-6, 6-7);  range ofR_s_= -0.04 to 0.01  (*p* = 1) for two comparisons  (day blocks 3-4, 4-5) |  |
| 4 (7 days) | Range of R_s_= 0.14 to 0.41  **(*p <*0.01**) | R_s_ = 0.28  **(*p <* 0.001)**for one comparison(day block 3-4);  R_s_ = 0.004  *(p* = 1) for one comparison  (day block 1-2);  R_s_ = -0.16  *(p* = 1) for one comparison  (day block 2-3) |  |
| 2 (14 days) | R_s_ = 0.41  **(*p <*0.001**) | R_s_= -0.07  (*p =* 1) |  |

**References**

1. Vázquez Diosdado JA, Barker ZE, Hodges HR, Amory JR, Croft DP, Bell NJ, Codling EA. Space-use patterns highlight behavioural differences linked to lameness, parity, and days in milk in barn-housed dairy cows. *PLoS One* (2018) **13**: doi:10.1371/journal.pone.0208424

2. Barker ZE, Vázquez Diosdado JA, Codling EA, Bell NJ, Hodges HR, Croft DP, Amory JR. Use of novel sensors combining local positioning and acceleration to measure feeding behavior differences associated with lameness in dairy cattle. *Journal of Dairy Science* (2018) **101**:6310–6321. doi:10.3168/jds.2016-12172

3. Patison KP, Swain DL, Bishop-Hurley GJ, Robins G, Pattison P, Reid DJ. Changes in temporal and spatial associations between pairs of cattle during the process of familiarisation. *Applied Animal Behaviour Science* (2010) **128**:10–17. doi:10.1016/j.applanim.2010.10.001

4. Rocha LEC, Terenius O, Veissier I, Meunier B, Nielsen PP. Persistence of sociality in group dynamics of dairy cattle. *Applied Animal Behaviour Science* (2020) **223**:104921. doi:10.1016/j.applanim.2019.104921

5. Hodges HR. An investigation of social structure in housed dairy cows. (2018) Available at: http://repository.essex.ac.uk/23324/ [Accessed July 11, 2020]

6. Boyland NK, Mlynski DT, James R, Brent LJN, Croft DP. The social network structure of a dynamic group of dairy cows: from individual to group level patterns. *Applied Animal Behaviour Science* (2016) **174**:1–10. doi:10.1016/j.applanim.2015.11.016
